# Unexpected invasion of miniature inverted-repeat transposable elements in viral genomes

**DOI:** 10.1186/s13100-018-0125-4

**Published:** 2018-06-18

**Authors:** Hua-Hao Zhang, Qiu-Zhong Zhou, Ping-Lan Wang, Xiao-Min Xiong, Andrea Luchetti, Didier Raoult, Anthony Levasseur, Sebastien Santini, Chantal Abergel, Matthieu Legendre, Jean-Michel Drezen, Catherine Béliveau, Michel Cusson, Shen-Hua Jiang, Hai-Ou Bao, Cheng Sun, Thomas E. Bureau, Peng-Fei Cheng, Min-Jin Han, Ze Zhang, Xiao-Gu Zhang, Fang-Yin Dai

**Affiliations:** 1 College of Pharmacy and Life Science, Jiujiang University, Jiujiang, China; 2 State Key Laboratory of Silkworm Genome Biology, Key Laboratory of Sericultural Biology and Genetic Breeding, Ministry of Agriculture, Southwest University, Chongqing, China; 3 School of Life Sciences, Chongqing University, Chongqing, 400044 China; 4 Clinical Medical College, Jiujiang University, Jiujiang, China; 5 Dipartimento di Scienze Biologiche, Geologiche e Ambientali, Università di Bologna, Bologna, Italy; 6 Unité de Recherche sur les Maladies Infectieuses et Tropicales Emergentes (URMITE), Aix-Marseille University, UM63, CNRS 7278, IRD 198, INSERM 1095, Institut Hospitalo-Universitaire (IHU)-Méditerranée Infection, AP-HM, 19-21 Boulevard Jean Moulin, 13385 Marseille, France; 7 Aix-Marseille University, Centre National de la Recherche Scientifique, Information Génomique and Structurale, Unité Mixte de Recherche 7256 (Institut de Microbiologie de la Méditerranée, FR3479), 13288 Marseille Cedex 9, France; 8 Institut de Recherche sur la Biologie de l’Insecte, CNRS UMR 7261, Université François-Rabelais de Tours, UFR Sciences et Techniques, 37200 Tours, France; 9 Laurentian Forestry Centre, Canadian Forest Service, Natural Resources Canada, Quebec, Canada; 10 Institute of Apicultural Research, Chinese Academy of Agricultural Sciences, Beijing, China; 11 Department of Biology, McGill University, Montréal, Quebec, Canada; 12 Poyang Lake Eco-economy Research Center, Jiujiang University, Jiujiang, China

**Keywords:** Virus, MITEs, Autonomous partner, Horizontal transfer, Genome evolution

## Abstract

**Background:**

Transposable elements (TEs) are common and often present with high copy numbers in cellular genomes. Unlike in cellular organisms, TEs were previously thought to be either rare or absent in viruses. Almost all reported TEs display only one or two copies per viral genome. In addition, the discovery of pandoraviruses with genomes up to 2.5-Mb emphasizes the need for biologists to rethink the fundamental nature of the relationship between viruses and cellular life.

**Results:**

Herein, we performed the first comprehensive analysis of miniature inverted-repeat transposable elements (MITEs) in the 5170 viral genomes for which sequences are currently available. Four hundred and fifty one copies of ten miniature inverted-repeat transposable elements (MITEs) were found and each MITE had reached relatively large copy numbers (some up to 90) in viruses. Eight MITEs belonging to two DNA superfamilies (*hobo/Activator/Tam3* and *Chapaev–Mirage–CACTA*) were for the first time identified in viruses, further expanding the organismal range of these two superfamilies. TEs may play important roles in shaping the evolution of pandoravirus genomes, which were here found to be very rich in MITEs. We also show that putative autonomous partners of seven MITEs are present in the genomes of viral hosts, suggesting that viruses may borrow the transpositional machinery of their cellular hosts’ autonomous elements to spread MITEs and colonize their own genomes. The presence of seven similar MITEs in viral hosts, suggesting horizontal transfers (HTs) as the major mechanism for MITEs propagation.

**Conclusions:**

Our discovery highlights that TEs contribute to shape genome evolution of pandoraviruses. We concluded that as for cellular organisms, TEs are part of the pandoraviruses’ diverse mobilome.

**Electronic supplementary material:**

The online version of this article (10.1186/s13100-018-0125-4) contains supplementary material, which is available to authorized users.

## Background

Transposable elements (TEs) are DNA fragments that can move from one place to another in their host genomes, often resulting in their own amplification in the process [1]. DNA transposons represent a specific group of TEs, and they can be classified as either “autonomous” or “non-autonomous” elements. Autonomous transposons can transpose by themselves, while non-autonomous elements require enzymes encoded *in trans* by autonomous elements to be mobile [2]. Miniature inverted–repeat transposable elements (MITEs) are non-autonomous elements typically showing high copy numbers and length homogeneity [3, 4]. MITEs are often flanked by terminal inverted repeats (TIRs) and produce a short target site duplication (TSD) upon integration. As a group, MITEs form several superfamilies classified according to sequence similarity between their TIRs or TSD and those of autonomous partners^3^. It has been shown that MITEs play important roles in eukaryotic evolution, including an increase in genome size, formation of new genes, and the regulation of gene expression [5–8].

The vertical inactivation theory predicts that TEs will not escape the final fate of an inevitable vertical extinction in their host genomes due to elimination or inactivation [9, 10]. However, two factors may promote the long-term persistence of TEs in organism genomes. First, TEs have the capacity to be introduced into a new genomic background via horizontal transfers (HTs) [11], and second, autonomous DNA transposons can generate numerous defective elements (or MITEs) that evade the host defense system, either because of their small in size (< 600-bp) and/or because they fail to trigger the host repression response [2]. MITEs have been found in numerous eukaryotes including representatives of plants, fungi, protozoans and metazoans [2–5, 8]. In contrast, only one MITE family with a substantial number of copies has been reported from the giant virus *P. salinus* [6]. To the best of our knowledge, the amount and impact of the canonical TEs (for example, typical Class I or Class II TEs) on the evolution of viruses remains largely unknown. All TEs reported so far in viral genomes, apart from one *Tc1/mariner* member recently discovered in the genome of *Pandoravirus salinus* [6], display very low copy numbers (usually one or two) [12–19]. This has been interpreted as suggesting that viruses simply act as vectors for horizontal transfer (HT) of specific TEs with no clear impact on viral genomes themselves. To investigate the impact of TEs on viral genome evolution, a large-scale systematic analysis was therefore required. For the present study, a comprehensive survey of MITEs was performed through a careful examination of all latest available viral genome sequences. We focused on MITEs because their small size may allow a long-term persistence in diverse lineages through evasion of the host defense system^2^ and because a substantial number of one MITE belonging to the *Tc1/mariner* superfamily has been found in the genome of a giant virus, *P. salinus* [6].

## Results

### Discovery and characterization of MITEs in viruses

To scan viral genomes for MITEs, we characterized MITEs *do novo* in the most recent publicly available database of viral genomes (5170 viruses) from viruSITE [20] as of May 2016 using MITE-hunter [21]. Ten candidate MITEs were retrieved and were classified into four superfamilies of DNA transposons (Additional file 1: Table S1). These excluded twelve elements whose TSD could not be determined (Additional file 2: Table S2). Boundaries of all the MITE candidates could be well-defined through alignment of multiple full-length copies along with their flanking sequences (Additional file 3: Figure S1a). These repeats shared all features reported for MITEs (3), including a small size (116–422-bp), TIRs, 2–8-bp TSDs, stable secondary structure, lack of coding capacity and high size homogeneity (Additional file 1: Table S1 and Additional file 3: Figure S1a,d). *Submariner-NA* or *Submariner* (see Methods for details on nomenclature) had previously been reported [6], but the remaining nine MITEs are reported here for the first time in viruses. Sequences of all MITEs and their autonomous elements identified in this study have been deposited in Repbase [22].

**Fig. 1:**
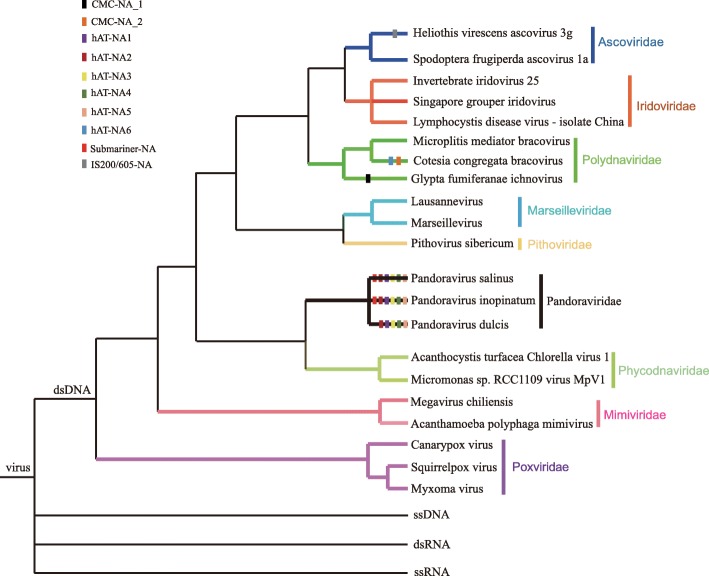
The schematic representation of phylogenetic relationships of viruses. The presence of MITEs in viral genomes is shown using rectangles. Species abbreviations were shown in Additional file 1: Table S1

**Fig. 2:**
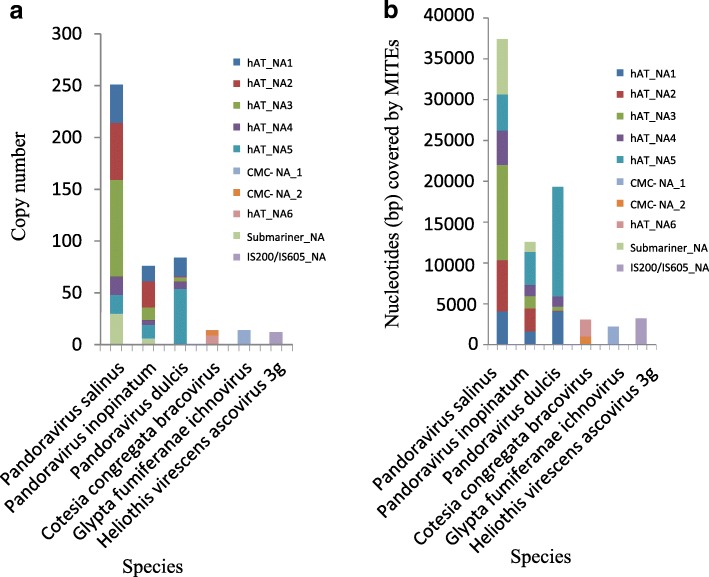
Copy number and content of ten MITEs in the viral genomes. **a** Comparison of copy numbers of ten MITEs in studied viruses. **b** Amount of nucleotide covered by each MITE in five viral genomes

Our survey revealed that MITEs were present in the genomes of viruses belonging to the *Ascoviridae*, *Polydnaviridae* (PDV) and *Pandoraviridae* families (Fig. 1). The number of MITEs also displayed important differences among viral genomes. For example, only one MITE (*CMC-NA_1*) was found in the genome of *Glypta fumiferanae ichnovirus* (GfIV), whereas six MITEs (*Submariner-NA*, *hAT-NA1*, *hAT-NA2*, *hAT-NA3*, *hAT-NA4*, *hAT-NA5*, and *hAT-NA6*) were detected in the genomes of the three known pandoraviruses (Fig. 1). Nucleotide genetic distance between each MITE copy and the consensus sequence varied from 0.06 to 0.4 (Additional file 3: Figure S1e). Meanwhile, the distribution of pairwise divergence among MITEs usually overlap, suggesting that these MITEs might amplify at similar time in these viral genomes. Comparisons of copy numbers and contents of MITEs between PDV and the *Pandoraviridae* showed that MITEs had experienced the most successful amplification in *Pandoravirus salinus*, the virus with the largest genome characterized to date [23]. Here, MITEs generated up to 251 copies, contributing with about 37.4-kb of DNA and constituting almost 1.5% of the viral genome (Fig. 2). This proportion of MITEs is even higher than that assessed for many insect and plant genomes [5, 8].

To determine whether MITEs identified in viruses in this study have been experienced transposition, paralogous empty sites (i.e., not containing MITEs) were searched in viral genomes using the flanking sequences (50 or 100-bp on either side) of all full-length elements as queries. One paralogous empty site of *CMC-NA_1* was found in the genome of GfIV, suggesting that *CMC-NA_1* had experienced transposition activity in this virus (Additional file 3: Figure S1b). Alternatively, this result might be explained by host-to-virus transposition into only one of the paralogous sites. *CMC-NA* The six MITEs discovered in the genomes of the three known pandoraviruses (Fig. 1 and Additional file 1 Table S1) enabled investigation of their presence/absence polymorphism at viral orthologous genomic sites using synteny and collinearity analyses. The analyses showed that these MITEs were widely distributed in syntenic and collinear regions of pandoraviruses, and that 262 orthologous genomic sites were devoid of MITEs (i.e., orthologous empty sites) (Additional file 4: Figure S2). These results provided strong evidence that MITEs had been transpositionally active after divergence of these pandoraviruses, contributing to their genetic diversity. Our results also showed that some MITE copies shared at seven orthologous sites between different viruses. For *hAT_NA1* and *hAT_NA2*, their copies were found at two and one orthologous sites shared by *P. salinus* and *P. dulcis*, respectively. For *hAT_NA4*, its copies were found at one orthologous site shared by *P. salinus* and *P. inopinatum*. For *hAT_NA5*, its copies were present at two orthologous sites shared by *P. salinus* and *P. dulcis* and also found at one orthologous site shared by *P. salinus* and *P. inopinatum*). This might imply that such MITEs have been conserved since the divergence of these viruses. Alternatively, it indicate that transposition might occur independently twice at the same locus in difference viruses.

### Putative autonomous partners for MITE transposition

**Fig. 3:**
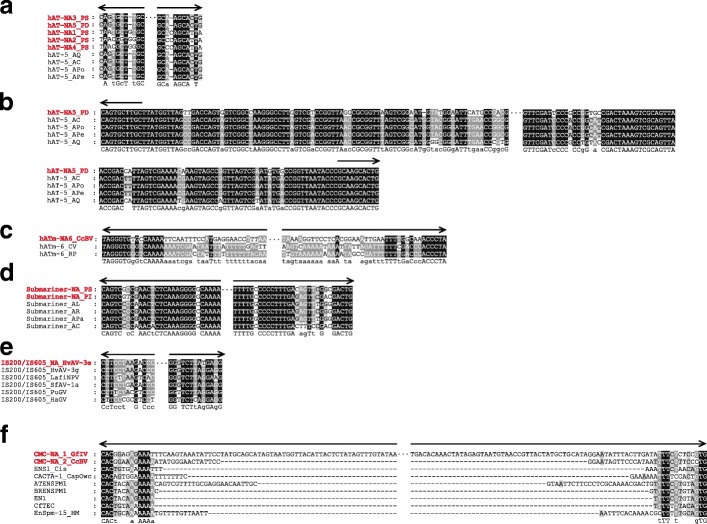
Multiple alignments of the 5′ and 3′ terminal sequences of *hAT-NA1* to *hAT-NA5* (**a** and **b**), *hATm-NA6* (**c**), *Submariner-NA* (**d**), *IS200/IS605_NA* (**e**) and *CMC-NA* (**f**) with their corresponding potential autonomous elements. MITEs identified in viruses are shown using red color. Species abbreviations are shown in Additional file 1: Table S1. *CfTEC* was obtained from the previous report [26], and the rest of the *CACTA* transposons were retrieved from Repbase [20]. TIRs of *hATm-6_RP* and *hATm-6_CV* are 417 bp and 575 bp, respectively. Therefore, only 40 bp of both termini of these transposons are shown using arrows

MITEs have no capacity of transposing and need to borrow the transpositional machinery from their master elements (2). Generally, the sequence similarity of MITEs and DNA transposon responsible for their transposition is restricted to TIRs. Therefore, we investigated potential autonomous elements of these MITEs in both viral and cellular host genomes (e.g. *Acanthamoeba castellanii* for pandoraviruses) using homology analysis. Autonomous DNA transposons potentially involved in the spread of eight MITEs were discovered in cellular hosts (or species related to their hosts) (Fig. 3 and Additional file 1: Table S1). We found that these autonomous elements had all the hallmarks of the corresponding superfamilies. For example, both *hAT-5* and *hATm-6* were flanked by 8-bp TSD and encoded an intact transposase with a conserved dimerization domain (PF05699) at the C-terminus, characteristic features of the *hAT* superfamily [24]. Interestingly, *hAT-5* was identified in four amoebas (*A. castellanii*, *A. polyphaga*, *A. pearcei* and *A. quina*), three of which contained *hAT-5* sharing 100% sequence identity at the nucleotide level, encoding a 620-amino-acid (aa) transposase (nucleotide positions: 279–2141) without disruption by premature stop codons. These data might suggest that *hAT-5* is still active in the amoeba hosts of pandoraviruses. Phylogenetic analysis of *hAT* transposases showed that *hAT-5* was a member of the *Ac* family (Additional file 5: Figure S3a). Although more than 74 *hATm* closely related to *hATm-6* have been deposited in Repbase [22], knowledge about these transposons remains relatively limited. Our results suggested that *hATm-6* might represent a fourth family of *hAT* transposons. First, the *hATm* family appears to be divergent from the other three reported *hAT* families (*Ac*, *Buster* and *Tip*) [24, 25] and forms a well supported clade, with bootstrap values of 100% (Additional file 5: Figure S3a). Second, we confirmed that 28 members of the *hATm* family had well-defined boundaries and that integration of these transposons generated 8 or 9-bp TSDs (Additional file 5: Figure S3b and Additional file 6: Table S3). Third, multiple alignments of their terminal sequences showed that *hATm* transposons were flanked by the conserved 5′-TAGGGTG and CACCCTA-3′ termini (Additional file 5: Figure S3c). We therefore propose to establish a new family within the *hAT* superfamily, designated *hATm* [22].

The *hAT-5* transposons identified in four amoebas had short TIRs (10 bp) that were identical to the terminal sequences of *hAT-NA3* and *hAT-NA5* (Fig. 3a and b, Additional file 1: Table S1). Similarly, we showed that 5 to 8 out of 10 nucleotides of *hAT-5* TIRs were identical to the termini of *hAT-NA1*, *hAT-NA2* and *hAT-NA4* (Fig. 3b). This level of TIR similarity between MITEs and their autonomous partners is typical [26]. We also observed that the sequence identity between *hAT-NA5* and *hAT-5* was not limited to the TIRs but also encompassed parts of the internal sequences of *hAT-5* (Fig. 3a). This result suggested that *hAT-NA5* was an internal deletion derivative of *hAT-5*. In view of the high level of similarity reported here for TIRs and TSD, *hAT-5* was likely responsible for the transposition of the five MITEs identified in pandoraviruses.

We also noticed similarity of termini (13/15 nucleotides) between *hATm-NA6_CcBV* and two autonomous elements from *Cotesia vestalis* and *Rhodnius prolixus* (Fig. 3c). Interestingly, autonomous DNA transposons sharing the same terminal sequences (5′-TAGGGTG and CACCCTA-3′) with that of *hATm-NA6_CcBV* were also present in the genomes of *Cotesia congregata bracovirus* (CcBV) (GenBank accession number MF689003) as well as in its insect host, *C. congregata* (Additional file 2: Table S2 and Additional file 5: Figure S3c). Analysis of its transposase showed that both autonomous elements had encoded a relatively recent function protein which suggests transposition activity occurred in the wasp genome impacting the virus.

Sun et al. [6] reported the presence of an autonomous partner of *Submariner-NA_PS* in the pandoravirus amoebal host *A. castellanii*. Here, we found that this autonomous element was also present in three other amoebas, *A. lugdunensis*, *A. rhysodes* and *A. palestinensis* (Additional file 1: Table S1). Our results showed that the autonomous partner discovered in *A. rhysodes* seemed to have a functional transposase, which encoded a 375-aa protein (nucleotide positions:339–1466) and is similar in size to other active transposases of *Tc1/mariner* transposons [27]. Moreover, the intact DDE signature, a central catalytic domain of *Tc1/mariner* transposases, was also identified in the *A. rhysodes* transposase sequence. All the above autonomous transposons included 29-bp TIRs that displayed high sequence identity with TIRs of *Submariner-NA* (Fig. 3d), suggesting that they might be involved in the transposition of *Submariner-NA*.

In the case of *IS200/IS605_NA*, its autonomous elements were found in two ascovirues and three viruses belonging to the family of Baculoviridae (Additional file 1: Table S1). *IS200/IS605_NA* are flanked by 10 bp TIRs, which are identical to those of its autonomous elements (Fig. 3d). However, these autonomous elements only encoded a protein of unknown function TnpB, and no transposase (TnpA) was found. Although no autonomous partner of *CMC-NA_1* and *CMC-NA_2* was found in the viral and cellular host genomes, sequence similarity was observed among TIRs of *CMC-NA_1*, *CMC-NA_2* and the reported *CACTA* transposons (Fig. 3f). In particular, 12/15 nucleotides of TIRs of *ENS1_Cis*, a *CACTA* transposon described in the transparent tunicate *Ciona savignyi* [22, 28], was identical to the TIRs of *CMC-NA_1_GfIV*. In addition, both *ENS1_Cis*, *CMC-NA_1* and *CMC-NA_2* shared the invariable 5′-CAC and GTG-3′ termini and created a 2-bp TSD (Additional file 3: Figure S1a,b). Thus, our results suggests that *CMC-NA_1* and *CMC-NA_2* are two members of the *CACTA* transposons, which are affiliated to a larger “megafamily” known as *CMC* for *Chapaev–Mirage–CACTA* [29].

### Horizontal transfers of MITEs between viruses and their hosts

**Fig. 4:**
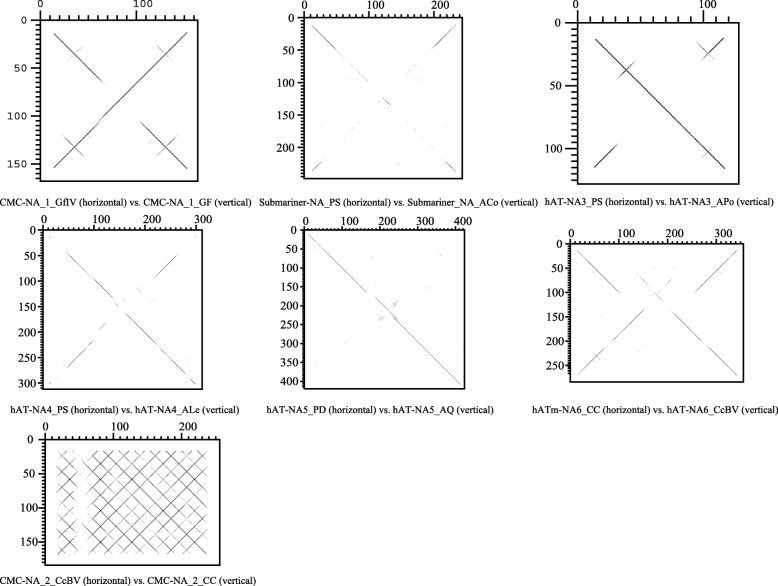
Dotplot comparsions of seven MITEs involved in HTs between viruses and its potential hosts. Species abbreviations were shown in Additional file 1: Table S1, and multiple alignments of these MITEs were indicated in Additional file 9: Figure S5

To identify the possible HTs of MITEs between viruses and their cellular hosts or closely related species, each MITE found in viruses was used as a query to search against cellular genomes. In the case of *CMC-NA_1*, *CMC-NA_2*, *Submariner-NA*, *hAT-NA3*, *hAT-NA4*, *hAT-NA5* and *hATm-NA6*, these searches yielded a number of significant hits (score ≥ 123, *e*-value ≤ 3 × 10^− 11^). *CMC-NA* and *hATm-NA6* were distributed in two and three wasps, respectively (Additional file 1: Table S1). The remaining four MITEs were found in eight amoebas. The boundaries of these MITEs could be determined in at least one cellular genome, revealing that they created a TSD upon insertion similar to that of MITEs found in viruses (Additional file 3: Figure S1 and Additional file 7: Figure S4). Almost all sequences presented the characteristics of non-autonomous elements, namely short length and lack of coding capacity (Additional file 1: Table S1). The alignment revealed sequence identity from 63.7 to 96.9% across the whole length of seven MITEs between any two species (Fig. 4, Additional file 8: Table S4 and Additional file 9: Figure S5). This high sequence identity strongly suggests that they were acquired through HTs. To rule out the possibility that the latter conclusion is compromised because based on accidental DNA cross-contamination artifacts, we conducted two additional analyses. First, MITEs computationally identified in the viral genomes were confirmed using PCR amplification and sequencing of PCR products (GenBank accession numbers MF576508-MF576521). Second, we plotted the coverage of six MITEs plus 1000 bp before and after their genomic positions on the genomes of *P. salinus* and *P. dulcis* and found no significant drop in coverage (Additional file 10: Figure S6).

**Fig. 5:**
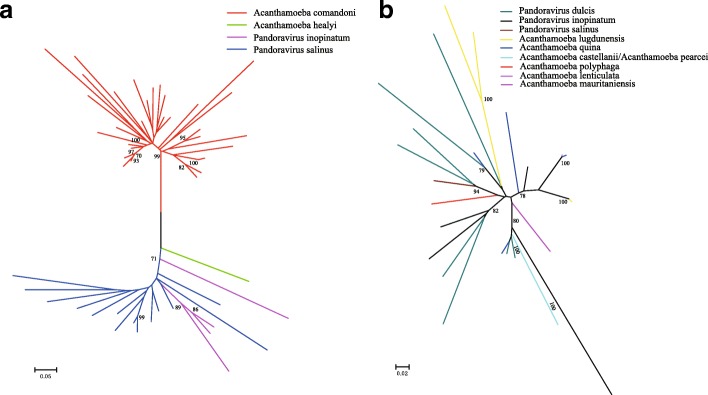
The phylogenetic relationship of two MITEs from viral and host genomes. **a** A phylogenetic tree of full-length copies of *Submariner-NA* elements from pandoraviruses and their host amoeba. **b** Phylogeny of full-length copies of *hAT-NA5* elements from pandoraviruses and their host amoeba. Clade credibility values (> 70%) are shown at each node. Each intact copy from different species is indicated using distinguishable color lines. Because *hAT-NA5* from *A. castellanii* and *A. pearcei* are identical, and they are displayed using fluorescent green lines

Next, we investigated the relationship between MITEs found in viral and host genomes. A phylogenetic tree of full-length copies of *Submariner-NA* elements indicated that pandoravirus elements formed a monophyletic clade (Fig. 5a), indicating that it was a single ancestral transfer, more likely from host to virus, followed by spread of the MITEs within viral genomes. We should also notice that this result can also be due to multiple host-to-virus HT from one or more hosts that have not been sequenced yet. In contrast, copies of the rest six MITEs from viruses intermingled with host elements in the phylogenies was indicative of possibly multiple HT events from a single ancestor TE into these species (Fig. 5b and Additional file 11: Figure S7). Encapsidated PDV genomes comprise multiple dsDNA circular segments (often referred to as “circles”), linear copies of which reside in the genomes of wasp hosts. The latter condition facilitates movement of TEs from wasp DNA to the integrated “proviral” PDV genome. This is apparently what happened in the case of the *CMC-NA_1*, *CMC-NA_2* and *hATm-NA6* found in the genomes of PDVs, as nearly identical copies of these TEs were observed in wasp DNA near proviral genome segments (Additional file 9: Figure S5b,c,g). Interestingly, no *CMC-NA_1* were found (data not shown) in the genome of species closely related to GfIV, *Apophua simplicipes ichnovirus* (AsIV) [30], an observation that suggests GfIV acquired *CMC-NA* from the genome of *G. fumiferanae* as opposed to a viral ancestor of both GfIV and AsIV. With respect to *CMC-NA_2* and *hATm-NA6* from bracoviruses, they similarly appear to have originated from the genomes of their braconid host wasps, as we failed to detect them in nudiviruses, the viral progenitor of bracoviruses [31].

## Discussion

Here, we reported on the successful invasion of virus genomes by additional nine MITEs, eight of which belonging to the *CMC* and *hAT* superfamilies were for the first time identified in viruses, further expanding the organismal scope of these two superfamilies. The *hAT* superfamily represents a very large and diverse group of DNA transposons widely distributed in the cellular world and the *hAT* transposons can also be domesticated to become important functional genes in eukaryotes [24, 25]. Here, our results clearly indicated that the *hAT* transposons were remarkable and reached high copy numbers in some DNA viruses (Fig. 2). This is in agreement with the capacity of *hAT* elements to transpose efficiently in eukaryotes [32]. A phylogenetic tree of *hAT* transposases and sequence analyses clearly supported the recognition of a fourth family (Additional file 5: Figure S3), which we here named *hATm*, present in a wide range of organisms.

There are six known families of giant viruses infecting *Acanthamoeba* species: *Mimiviridae*, *Pandoraviridae*, *Marseilleviridae*, *Faustovirus*, *Pithoviridae*, and Mollivirus [33], with genome sizes ≥ 500 kb. The genome of *P. salinus*, 2.5-Mb in size [23], is the largest genome of all sequenced viruses and is even larger than the genomes of some bacteria such as *Tremblaya* [34] and *Rickettsia* [35]. Therefore, the discovery of pandoraviruses with genomes up to 2.5-Mb emphasizes the need for biologists to rethink the fundamental nature of the relationship between viruses and cellular life. Because there are large differences in genome size – even among giant viruses – the factors driving the evolution of these genomes have been the subject of intense debate and research. Many studies showed that genome evolution of giant viruses was related to successive steps of gene duplication, gene transfer and amplification of other types of repeated sequences (e.g. *IS607* from the bacteria and archaea) [23, 36–38]. MITEs were observed here to be most abundant in pandoraviruses, a condition that may have played a role in genome expansion of these viruses. This result is consistent with the earlier observations made on plants and insects [5, 8]. Interestingly, all *hAT* transposons identified in pandoraviruses have very similar termini (Fig. 3a), suggesting that they are derived from some close related progenitor elements. Generally, TEs reported in the viral genomes had only one or two copies. In contrast, we found that MITEs in every case have expanded within the ascovirus, PDV and pandoravirus genomes (Additional file 1: Table S1). In addition, we should note that other undetermined twelve repeat elements were found in other seven families of ds DNA viruses (Additional file 2: Table S2). Meanwhile, almost all these repeat elements have amplified in viral genomes. Together, our results may suggest that TEs have played important roles in the genome evolution of ds DNA viruses.

MITEs have been found both in ascovirus, PDVs and pandoraviruses, which may be related, in both cases, to low constraints on genome size, making it possible to accommodate insertions. Most viruses have a genome strictly limited in size, each single particle has to contain the whole set of genes required to support a new replication cycle and the size of genetic material incorporated strictly depends on physical capacity of the particles. This results in most virus genomes having high gene density. In such compact genomes, any insertion is likely to have a deleterious effect for the virus, which would result in its elimination by counter selection. By contrast, the capacity of giant viruses particles to incorporate DNA is high and the cumulative size of the packaged viral molecules of *Polydnaviridae* appears to be similar to that of “giant viruses” with over 800 kb for CcBV [39]. Moreover, PDVs are solely transmitted vertically as endogenous viruses of parasitic wasps, and there is no replication in infected cells. The genes involved in particle production reside permanently in the wasp genome and have been replaced in the DNAs packaged in the particles by virulence genes. These are introduced during wasp oviposition and are essential to alter parasitized host immune response and, more broadly, its physiology (metabolism, development), favouring wasp larvae development. Sequencing of encapsidated PDV genomes has shown that, resulting from these particular constraints, the coding density is unusual low for a virus, with about 70% of non coding sequence in the case of CcBV [40]. This may allow to maintenance of MITE insertions. Accordingly, several remnants of TEs have been previously identified in dsDNA circles packaged in the particles including retroelements *Dong* and *DIRS* and a large DNA transposon *Maverick* [12, 14]. PDV proviral segments from which circles are produced have been integrated for up to 100 million years in the wasp genomes [41] and, as such, they have been regularly the target of TE insertions including those of *hATm-NA6* and other MITEs reported here.

Unlike most other viral hosts, amoebae are usually infected by a variety of microorganisms through amoebal attack [42]. Free-living amoeba have been described as “melting pots” for genetic exchanges because of the stable coexistence of different parasites in amoeba [37, 38, 43, 44]. This condition increases possibilities HTs between the host and its parasites and among the different parasites within their amoebal host. In the present study, HTs of four MITEs have taken place between pandoraviruses and their amoebal host. To the best of our knowledge, this is the first convinced documented example of MITEs from giant viruses and their hosts being involved in HTs. Meanwhile, three addition HT cases of MITEs, probably from wasps to PDVs, were found. PDVs represent a highly unconventional group of DNA viruses whose genomes have become integrated into the genome of their wasp hosts. This condition will be propitious to introduce MITEs from wasps to PDVs. The above results also provide evidence supporting that viruses could be served as vectors for transposon HTs. In contrast, the mechanisms of most HTTs have been lacking, although nearly three thousand cases of HTTs have been reported in organisms [45, 46].

Although the viral genomes, especially pandoraviruses, were found to be rich in MITEs, these TEs do not have the capacity to mediate their own transposition. From this observation, one question arises: how did MITEs amplify and colonize viral genomes. In this study, autonomous partners (*Submariner*, *hAT-5* and *hATm-6*) of seven MITEs were detected in viral hosts. Sequence similarity between MITEs and these autonomous elements are largely limited to their termini or TIRs (Fig. 2), which are usually the only requirement for transposase recognition of DNA transposons during the process of transposition^1^. This result implies that *Submariner*, *hAT-5* and *hATm-6* may be responsible for transposition of the MITEs we found in DNA viruses. Because *Submariner*, *hAT-5* and *hATm-6* appear to be still active in viral hosts, the stage is set for MITEs found in viruses to be amplified again in the future.

## Conclusions

Our discovery highlights that TEs contribute to shape genome evolution of pandoraviruses. We concluded that as for cellular organisms, TEs are part of the pandoraviruses’ diverse mobilome.

## Methods

### Identification and copy number determination of MITEs in viral genomes

We downloaded the latest publicly available genome assemblies of 5170 viruses from viruSITE [20] as of May 2016. MITEs from the above viral genomes were characterized de novo using MITE-hunter with default parameters [21]. Multiple sequence alignment (MSA) files generated by MITE-hunter were manually analyzed for boundaries and TIRs and TSD of potential TEs. Based on the characteristics of MITEs [3], TEs that meet the following criteria are considered as a false-positive: (1) Length of TEs longer than 800 bp; (2) TEs with no TIRs or TSD; (3) Compound TEs. The consensus sequences of candidate MITEs were reconstructed using DAMBE with default parameters [47], and were served as a custom repeat library to pass through a local version of RepeatMasker (http://www.repeatmasker.org) using the search engine RMBlast to calculate the MITE copy number. Fragments shorter than 80 bp were manually excluded. The secondary structure of MITEs was predicted using mFold [48]. Pairwise distance between each copy and the consensus sequence was calculated using MEGA 4 (Pairwise deletion, Maximum composite likelihood model) [49]. Paralogous empty sites of candidate MITEs were found using the method previously described [17].

### Identification and characterization of candidate MITE-related sequences and putative autonomous partners in their potential host genomes

Based on similar TIRs and TSD with those of known DNA transposons [3], ten of candidate MITEs identified in viruses could be classified into the superfamilies. We noticed that these ten MITEs resided in viruses, which infect insects or amoebas. Therefore, we looked for these MITE-related sequences in the viral hosts. We used the consensus sequences or representatives of these ten MITEs as queries to BLASTn against the publicly available genome sequences of 227 insects and 15 amoebas as well as the National Center for Biotechnology Information (NCBI) Nucleotide collection database, and all obtained hits were manually checked. If MITEs found in one species were present in less than five full-length copies, which make us difficult to reconstruct reliable consensus sequences, the best match of the queries was used to calculate its copy number and construct multiple alignments together with MITEs from other species. To test for HTs, MITEs were considered to be present in one viral host if one transposon showed ≥ 60% similarity to the consensus sequences at the nucleotide level and over ≥ 60% of their length. To identify their potential autonomous partners, all significant hits ranging from 1000 bp to 10-kb bearing some similar sequences with those of eight MITEs were retrieved from viral and their host genomes. These longer elements were characterized using the following four methods: (1) TIRs were predicted using fastPCR [50]; (2) Open reading frame was analyzed using FGENESH [51] or GENSCAN (http://genes.mit.edu/GENSCAN.html); (3) The NCBI Conserved Domain Database (CDD) was employed to identify their putative functional domains; (4) Their nucleotide or protein sequences were used as queries to search against Repbase [22].

### Nomenclature

In this study, both several MITEs and their autonomous elements were found in the viral hosts. To avoid confusion, each MITE and the corresponding autonomous element were named *X-NA#* (where X represents a superfamily, NA represents nonautonomous elements, # is a letter representing the corresponding numbers of transposons) and *X-#*, respectively (Additional file 1: Table S1). We also noted that both a MITE identified in *P. salinus* and its autonomous element found in its cellular host were named *Submariner*_*PI* and *Submariner*_*Ac1/Submariner*_*Ac2*, respectively [6]. We have now found this MITE and the autonomous element were widespread in viruses and their hosts (Additional file 1: Table S1). For these reasons, we decide to introduce the name *Submariner-NA* for this MITE and *Submariner* for its corresponding autonomous element.

### Sequence analysis

To identify *hATm*-like elements in other species, we carried out BLASTp and tBLASTn searches using the predicted *hATm* transposase sequence from the blood sucking bug *R. prolixus* as a query against the NCBI Non-redundant protein and Nucleotide collection database, respectively. All significant hits with an identity ≥ 30% and coverage ≥ 60% with an *e*-value ≤ 5 × 10^− 20^ were obtained [25]. The detail information is shown in Additional file 12: Table S5 and Additional file 13: Table S6. Copies of all retrieved sequences were extracted with 5000 bp flanking sequences from their host genomes to determine the boundary as well as TSD. Sequence logos were created by WebLogo [52] using 30 nt (15 nt upstream and 15 nt downstream) of their flanking sequences including TSD.

Transposase amino acid sequences from 102 *hAT* elements have been aligned using T-Coffee [53], with *expresso* mode (structural alignment). Ambiguous/uninformative alignment positions were, then, removed through Noisy v. 1.5 (Dress et al., 2008), with *cutoff* value = 0.8 and *distance* = GTR. A final alignment of 2210 amino acids was used to perform phylogenetic analyses. The best-fit model of evolution was estimated through ProtTest v.3 [54] based on BIC score, and the LG model with gamma distribution (+G) was selected as best substitution model. Maximum Likelihood tree was obtained through RAxML v. 8 [55], using 500 rapid bootstrap replicates for node support.

To determine the relationship of MITEs found in viruses and their hosts, multiple alignments of full-length copies of one MITE from one species were created using MUSCLE [56]. All ambiguous sites were removed, then a neighbor-joining tree was built using MEGA 4 [49] (Pairwise deletion, Maximum composite likelihood model, 1000 bootstrap replicates). Clade credibility values (> 70%) are shown at each node. Fifty full-length copies were randomly selected to include in this analysis for one MITE with more than fifty full-length copies in one species.

To assess misassemble (chimera) regions around MITEs, the PacBio reads that were used to assemble the *P. salinus* and *P. dulcis* genomes were mapped on the assembled genomes using BLASR [57]. The coverage was computed as the number of reads for each genomic position and was plotted for 2-kb fragments centered at the position of each MITE site.

Gene sequences of *P. dulcis*, *P. salinus* and *P. inopinatum* as well as a gff file for positions of predicted genes were downloaded from NCBI. Then, pairwise comparison of gene sequences from three pandoraviruses were performed using BLASTp [58], and gene synteny and collinearity were analyzed using MCScanX [59]. MITEs were mapped on the genomes of three pandoraviruses according to each genomic position of copies.

### Polymerase chain reaction (PCR) and sequencing

To confirm the presence of MITEs and the potential autonomous element computationally identified in the viral genomes, we designed the PCR primers using the flanking sequences of these MITEs. Primers were listed in Additional file 14: Table S7. For *P. inopinatum*, genomic DNA was extracted using EZ1 DNA Tissue Kit (QIAGEN) through automated extraction system: EZ1 Advanced XL (QIAGEN). PCR amplification was performed in a 25-μl volume including12.5 μl AmpliTaq Gold 360 Master Mix, 5 μl Pandoravirus genomic DNA, 5.5 μl H2O, 1 μl forward primer and 1 μl reverse primer. PCR runs were carried out with an initial denaturation step for 15 min at 95 °C followed by 39 cycles of denaturation at 95 °C for 30 s, annealing at 58 °C for 30 s, and elongation at 72 °C for 1 min, ending with a 5 min elongation step at 72 °C. Products of PCR were assayed using agarose gel electrophoresis, and were sequenced directly. For GfIV, viral DNA extraction was carried out as described previously [60, 61] and 13 ng of DNA was used for PCR amplification (in 1× PCR buffer) supplemented with 0.33 mM each deoxynucleoside triphosphate (dNTP), 0.25 μM each primer, and 1 unit of *Taq* DNA polymerase in a 30 μl reaction volume. The amplification was performed for 35 cycles consisting of 94 °C for 30 s, 55 °C for 30 s, and 72 °C for 1 min, with a final 5 min extension step at 72 °C. Five microliters per reaction was analyzed on a 1% agarose gel electrophoresis to confirm single amplicon before Sanger sequencing using the same primers used for amplification (Genome Sequencing and Genotyping platform of the CHUL, in Quebec City, Quebec, Canada). For CcBV, PCR reactions were performed on genomic DNA extracted from individuals using GoTaq (Promega, France) in a final volume of 25 μl containing 50 ng of male wasp genomic DNA, 25 U of *GoTaq*, 3 mM MgCl_2_ and 20 pmole of each specific primer with the following cycling conditions: 4 min of initial denaturation at 94 °C, followed by 35 cycles of denaturation at 94 °C for 40 s, primer hybridization at 58 °C for 40 s, extension at 72 °C for 60 s, and final extension at 72 °C for 10 min. PCR products were purified using adsorption columns and sequenced by Sanger technology by GATC Biotech (Germany) in both strands using the same forward and reverse primers as for PCR.

## Additional files

Additional file 1: **Table S1.** Characteristics of ten MITEs and its autonomous elements identified in this study. (DOC 144 kb)

Additional file 2: **Table S2.** Nucleotide sequences of twelve elements whose TSD could not be determined. (XLSX 11 kb)

Additional file 3: **Figure S1.** Characteristics of ten MITEs from the viral genomes. **A** Multiple alignments of full-length copies as well as the flanking sequences of each MITE. TSD is shown in red and the boundary is indicated using black shading. **B** One empty paralogous site of *CMC-NA_1_GfIV*. **C** Secondary structure of ten MITEs. **D** Length distribution of MITEs with more than 14 copies in one viral genome. **E** Nucleotide genetic distances between each MITE copy and the corresponding consensus sequences. Only MITEs with reliable consensus sequences in one virus were included in this analysis. Because more than 50% copies of *hAT-NA5_PD* were shorter than 50% length of the consensus sequence, and it was also excluded in this analysis. Species abbreviations of viruses were listed in Additional file 1: Table S1. (PDF 3703 kb)

Additional file 4: **Figure S2.** Gene synteny and collinearity of three Pandoraviruses and distribution of six MITEs on its genomes. **A** Circle plot showing patterns of synteny and collinearity of three Pandoraviruses. MITEs on the genomes of three Pandoraviruses are shown using different colored lines. **B** One example of the present of one copy of MITEs in orthologous genomic site of *P. salinus* and *P. dulcis* but absent in that of *P. inopinatum*. **C** One example of the absence of one copy of MITEs orthologous genomic site of *P. inopinatum* and *P. dulcis* but present in that of *P. salinus*. (PDF 431 kb)

Additional file 5: **Figure S3.** Phylogeny of three known families (*Buster*, *Ac* and *Tip*) of the *hAT* transposons with the *hATm* family and characteristics of TSD of two members of the *hATm* family. **A** Phylogenetic analysis of *hAT* transposases, performed through Maximum Likelihood method. Numbers at nodes represent bootstrap support after 500 replicates. Four members (*hAT5-AC*, *hAT5_AP*, *hAT5_APo* and *hAT5_AQ*) of the *Ac* family were found in this study. The detail information of the *hATm* transposons is listed in Additional file 1: Table S2. **B** TSD of *hATm-1_CuQ* and *hATm-1_BeT*. The results of sequence logos and empty paralogous site analysis indicated that *hATm-1_CuQ* and *hATm-1_BeT* created a 9-bp TSD upon insertion. **C** Multiple alignments of the 5′ and 3′ terminal sequences of 28 *hATm* transposons identified in this study. Because *hATm* transposons are generally flanked by long TIRs (e.g. TIRs of *hATm-6_RP* are 417 bp), and only 40 bp of both termini of these transposons were shown arrows. (PDF 1651 kb)

Additional file 6: **Table S3.** Target site duplications of *hATm* transposons. (DOC 59 kb)

Additional file 7: **Figure S4.** Determination of the boundary and TSD of seven MITEs identified in viral hosts or species related to their hosts. **A** Multiple alignments of full-length copies as well as the flanking sequences of each MITE. TSD is shown using red color and the boundary is indicated usingi black shading. **B** Empty paralogous sites of five MITEs identified in viral cellular hosts. (PDF 2219 kb)

Additional file 8: **Table S4.** Pairwise comparison of nucleotide sequence identity of seven MITEs involved in HTs between viruses and their cellular hosts or species closely related to their hosts. (DOC 68 kb)

Additional file 9: **Figure S5.** Multiple alignments of seven MITEs involved in HTs between viruses and its cellular hosts or species related to their hosts. (PDF 5206 kb)

Additional file 10: **Figure S6.** Mapping MITEs on the *P. salinus* and *P. dulcis* genomes. The 2 kb fragments coverage centered on each MITE copy including *hAT-NA1*, *hAT-NA2*, *hAT-NA3*, *hAT-NA4*, *hAT-NA5* and *Submariner-NA* for *P. salinus* (**A**) and *hAT-NA1*, *hAT-NA2*, *hAT-NA3*, *hAT-NA4*, *hAT-NA5* for *P. dulcis* (**B**) The blue horizontal line corresponds to zero coverage, the red line is the mean coverage and the green line is the median. (PDF 376 kb)

Additional file 11: **Figure S7.** Phylogenies of full-length copies of five MITEs involved in HTs between viruses and their hosts. (PDF 482 kb)

Additional file 12: **Table S5.** Number of significant hits of *hATm-6-RP* retrieved using BlastP and TBlastN Tools in NCBI and the percent average identity find with the query. (DOC 44 kb)

Additional file 13: **Table S6.** Detail information of significant hits of proteins related to *hATm-6-RP* retrieved using BlastP and TBlastN Tools in NCBI. (DOC 109 kb)

Additional file 14: **Table S7.** The detail information of primers to amplify MITEs and their potential autonomous partner in viruses. (DOC 43 kb)

## Abbreviations

CDD: Conserved Domain Database
*CMC*: *Chapaev–Mirage–CACTA*
dNTP: Deoxynucleoside triphosphate
GfIV: *Glypta fumiferanae ichnovirus*
HTs: Horizontal transfers
MITEs: Miniature inverted-repeat transposable elements
MSA: Multiple sequence alignment
NCBI: National Center for Biotechnology Information
PCR: Polymerase chain reaction
PDV: *Polydnaviridae*
TEs: Transposable elements
TIRs: Terminal inverted repeats
TSD: Target site duplication
