# Supplementary material for: Unexpected invasion of miniature inverted-repeat transposable elements in viral genomes
Source: Mob DNA. 2018 Jun 18;9:19. doi: 10.1186/s13100-018-0125-4 (PMC6004678; doi:10.1186/s13100-018-0125-4)
Supplement: Supplementary file 1 — Table S1. Characteristics of ten MITEs and its autonomous elements identified in this study. (DOC 144 kb) [file 13100_2018_125_MOESM1_ESM.doc]

Additional file 1: Table S1 Characteristics of ten MITEs and their autonomous elements of DNA transposons identified in this study

| Superfamily | TE names | Species | Length (bp) | Copy number | TSD | Tpase (aa) |
| --- | --- | --- | --- | --- | --- | --- |
| *CMC* |  |  |  |  |  |  |
|  | *CMC-NA_1* | *Glypta fumiferanae ichnovirus* (GfIV) | 139-166 | 14 | 2 bp | - |
|  |  | ***Glypta fumiferanae****^&^* **(GF)** | 156-166 | >3 | 2 bp | - |
|  |  | ***Microplitis demolitor* (MD)** | 84-172 | 162 | 2 bp | - |
|  | *CMC-NA_2* |  |  |  |  |  |
|  |  | *Cotesia congregata virus^#^* (CcBV) | 133-267 | 9 | ND | - |
|  |  | *Microplitis mediator bracovirus^&^* (MmBV) | 258 | ND | ND |  |
|  |  | *Glyptapanteles flavicoxis bracovirus^&^* (GfBV) | 174 | ND | ND |  |
|  |  | ***Cotesia congregata* (CC)** | 181 | ND | ND |  |
|  |  | ***Cotesia vestalis****^#^* **(CV)** | 80-238 | 250 | 2 bp | - |
|  |  | ***Microplitis demolitor* (MD)** | 80-296 | 8191 | 2 bp | - |
|  |  | ***Glyptapanteles flavicoxis****^&^* **(GF)** | 145 | ND | ND | - |
|  |  | ***Glyptapanteles indiensis****^&^* **(GI)** | 178 | ND | ND | - |
| *Tc1/mariner* |  |  |  |  |  |  |
|  | *Submariner-NA* | *Pandoravirus salinus* (PS)^*^ | 102-268 | 30 | TA | - |
|  |  | *Pandoravirus inopinatum^#^* (PI) | 87-250 | 6 | TA | - |
|  |  | ***Acanthamoeba comandoni* (ACo)** | 80-264 | 239 | TA | - |
|  |  | ***Acanthamoeba healyi****^#^* **(AH)** | 244 | 1 | TA | - |
|  | *Submariner* | ***Acanthamoeba lugdunensis*** *^#^* **(AL)** | 80-1379 | 10 | TA | 173 |
|  |  | ***Acanthamoeba rhysodes****^#^* **(AR)** | 1594 | >1 | TA | 375 |
|  |  | ***Acanthamoeba palestinensis****^#^* **(APa)** | 216-1593 | 7 | TA | 253 |
|  |  | ***Acanthamoeba castellanii****^#^* **(AC)**^*^ | 1604 | ND | TA | 382 |
| *IS200/IS605* |  |  |  |  |  |  |
|  | *IS200/IS605_NA* |  |  |  |  |  |
|  |  | *Heliothis virescens ascovirus 3e^#^* (HvAV-3e) | 89-527 | 12 | 4 bp | - |
|  |  | ***Heliothis virescens ascovirus 3g****^#^***(HvAV-3g)** | 1949-1955 | 5 | 4 bp | 608 |
|  |  | ***Spodoptera frugiperda ascovirus 1a****^#^* **(SfAV-1a)** | 1952-1954 | 2 | 4 bp | 606 |
|  |  | ***Lambdina fiscellaria nucleopolyhedrovirus****^#^***(LafiNPV)** | 1960 | 1 | ND | 608 |
|  |  | ***Helicoverpa armigera granulovirus****^#^* **(HaGV)** | 1963-1968 | 2 | 4 bp | 576 |
|  |  | ***Pseudaletia unipuncta granulovirus*** *^#^* **(PuGV)** | 1961 | 2 | 4 bp | 557 |
| *hAT* |  |  |  |  |  |  |
|  | *hAT-NA1* | *Pandoravirus salinus* (PS) | 95-118 | 37 | 8 bp | - |
|  |  | *Pandoravirus inopinatum* (PI) | 83-119 | 15 | 8 bp | - |
|  |  | *Pandoravirus dulcis* (PD) | 81-122 | 18 | 8 bp | - |
|  | *hAT-NA2* | *Pandoravirus salinus* (PS) | 88-127 | 55 | 8 bp | - |
|  |  | *Pandoravirus inopinatum* (PI) | 83-124 | 25 | 8 bp | - |
|  |  | *Pandoravirus dulcis^#^* (PD) | 97 | 1 | ND | - |
|  | *hAT-NA3* | *Pandoravirus salinus* (PS) | 104-138 | 93 | 8 bp | - |
|  |  | *Pandoravirus inopinatum* (PI) | 115-131 | 12 | 8 bp | - |
|  |  | *Pandoravirus dulcis*^#^ (PD) | 125-126 | 4 | ND | - |
|  |  | ***Acanthamoeba lugdunensis****^#^* **(AL)** | 103 | 1 | ND | - |
|  |  | ***Acanthamoeba polyphaga****^#^* **(APo)** | 107-126 | 2 | 8 bp | - |
|  | *hAT-NA4* | *Pandoravirus salinus* (PS) | 108-323 | 18 | ND | - |
|  |  | *Pandoravirus inopinatum*^#^ (PI) | 241-314 | 5 | ND | - |
|  |  | *Pandoravirus dulcis^#^* (PD) | 84-291 | 7 | ND | - |
|  |  | ***Acanthamoeba lenticulata****^#^* **(ALe)** | 104-312 | 5 | 8 bp | - |
|  |  | ***Acanthamoeba polyphaga****^#^* **(APo)** | 102-215 | 2 | ND | - |
|  |  | ***Acanthamoeba quina****^#^* **(AQ)** | 82-436 | 24 | 8 bp | - |
|  | *hAT-NA5* | *Pandoravirus salinus^#^* (PS) | 86-366 | 18 | ND | - |
|  |  | *Pandoravirus inopinatum^#^* (PI) | 96-419 | 13 | 8 bp | - |
|  |  | *Pandoravirus dulcis* (PD) | 85-428 | 54 | 8 bp | - |
|  |  | ***Acanthamoeba lenticulata****^#^* **(ALe)** | 293-375 | 2 | ND | - |
|  |  | ***Acanthamoeba lugdunensis****^#^* **(AL)** | 80-413 | 34 | ND | - |
|  |  | ***Acanthamoeba mauritaniensis****^#^* **(AM)** | 94-428 | 7 | 8 bp | - |
|  |  | ***Acanthamoeba polyphaga****^#^* **(APo)** | 103-429 | 12 | 8 bp | - |
|  |  | ***Acanthamoeba pearcei****^#^* **(APe)** | 80-420 | 46 | ND | - |
|  |  | ***Acanthamoeba quina****^#^* **(AQ)** | 82-419 | 48 | 8 bp | - |
|  |  | ***Acanthamoeba castellanii****^#^* **(AC)** | 415 | 1 | ND | - |
|  | *hAT-5* | ***Acanthamoeba castellanii****^#^* **(AC)** | 6082 | 1@ | 8 bp | 620 |
|  |  | ***Acanthamoeba polyphaga****^#^* **(APo)** | 6082 | 1@ | 8 bp | 620 |
|  |  | ***Acanthamoeba pearcei****^#^* **(APe)** | 6082 | 1@ | 8 bp | 620 |
|  |  | ***Acanthamoeba quina****^#^* **(AQ)** | 6427 | 1@ | ND | 623 |
|  | *hATm-NA6* | *Cotesia congregata bracovirus*^#^ (CcBV) | 80-282 | 5 | 8 bp | - |
|  |  | *Cotesia sesamiae Kitale bracovirus^&^* (CsKBV) | 355 | ND | ND | - |
|  |  | *Cotesia sesamiae Mombasa bracovirus^&^* (CsMBV) | 355 | ND | ND | - |
|  |  | *Cotesia vestalis bracovirus^&^* (CvBV) | 354 | ND | ND | - |
|  |  | *Cotesia plutellae polydnavirus^&^* (CpPV) | 339 | ND | ND | - |
|  |  | ***Glyptapanteles indiensis^&^* (GI)** | 354 | ND | 8 bp | - |
|  |  | ***Cotesia congregata*^&^ (CC)** | 354 | ND | 8 bp | - |
|  |  | ***Cotesia vestalis* (CV)** | 80-387 | 871 | 8 bp | - |
|  | *hATm-6* | ***Cotesia vestalis*^#^ (CV)** | 80-4737 | 1377 | 8 bp | 624 |
|  |  | ***Rhodnius prolixus*^#^ (RP)** | 83-4388 | 87 | 8 bp | 730 |

note: ND indicated that we could not determine its TSD, and - showed that these repeats were non-autonomous elements and did not encode a transposase.* indicated that *Submariner-NA* from *Pandoravirus salinus* and its autonomous element from *Acanthamoeba castellanii* were obtained from the previous study. *^#^* showed that MITEs in the genomes of these viruses had no more than five full-length copies or highly fragment copies, which make us difficult to construct the reliable consensus sequences. ^&^ indicated that this sequence was obtained from nt database of NCBI. @ showed that the sequence homology of *hAT-NA5* and *hAT-5* was not limited to the TIRs but also encompassed part of the internal sequences of *hAT-5* and the longest one of *hAT-5* was used to calculate the copy number in these species. MITEs identified in the viral hosts or closely related species was marked out using black bold, and autonomous elements present in viral hosts or closely related species were showed using red color. The characters in parentheses represented the species abbreviations.
