# Supplementary material for: Unexpected invasion of miniature inverted-repeat transposable elements in viral genomes
Source: Mob DNA. 2018 Jun 18;9:19. doi: 10.1186/s13100-018-0125-4 (PMC6004678; doi:10.1186/s13100-018-0125-4)

Additional file 3: Figure S1.

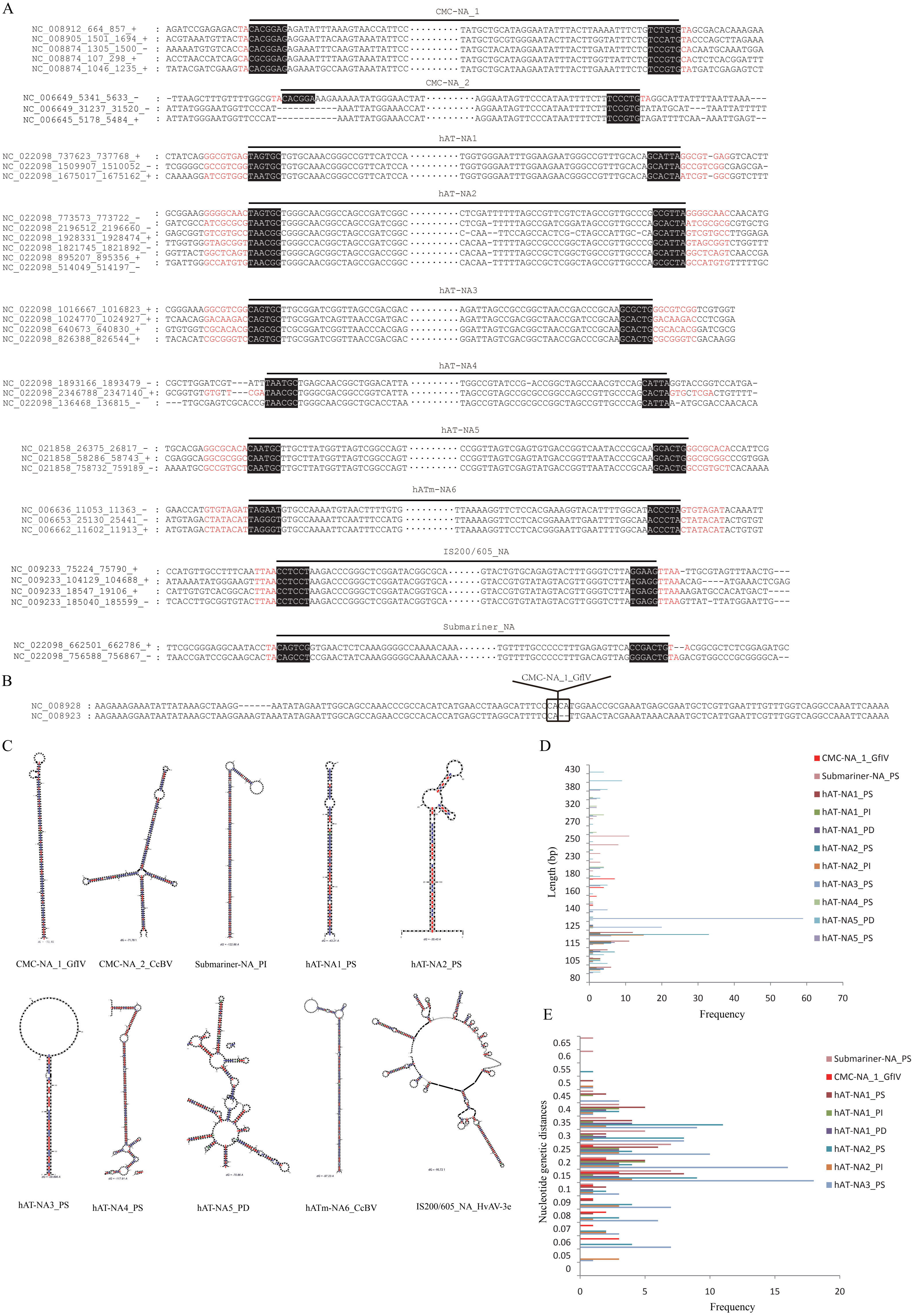

Supplement: Supplementary file 3 — Figure S1. Characteristics of ten MITEs from the viral genomes. A Multiple alignments of full-length copies as well as the flanking sequences of each MITE. TSD is shown in red and the boundary is indicated using black shading. B One empty paralogous site of CMC-NA_1_GfIV. C Secondary structure of ten MITEs. D Length distribution of MITEs with more than 14 copies in one viral genome. E Nucleotide genetic distances between each MITE copy and the corresponding consensus sequences. Only MITEs with reliable consensus sequences in one virus were included in this analysis. Because more than 50% copies of hAT-NA5_PD were shorter than 50% length of the consensus sequence, and it was also excluded in this analysis. Species abbreviations of viruses were listed in Additional file 1: Table S1. (PDF 3703 kb) [file 13100_2018_125_MOESM3_ESM.pdf]
