# Supplementary figures and images for: Unexpected invasion of miniature inverted-repeat transposable elements in viral genomes
Source: Mob DNA. 2018 Jun 18;9:19. doi: 10.1186/s13100-018-0125-4 (PMC6004678; doi:10.1186/s13100-018-0125-4)

A

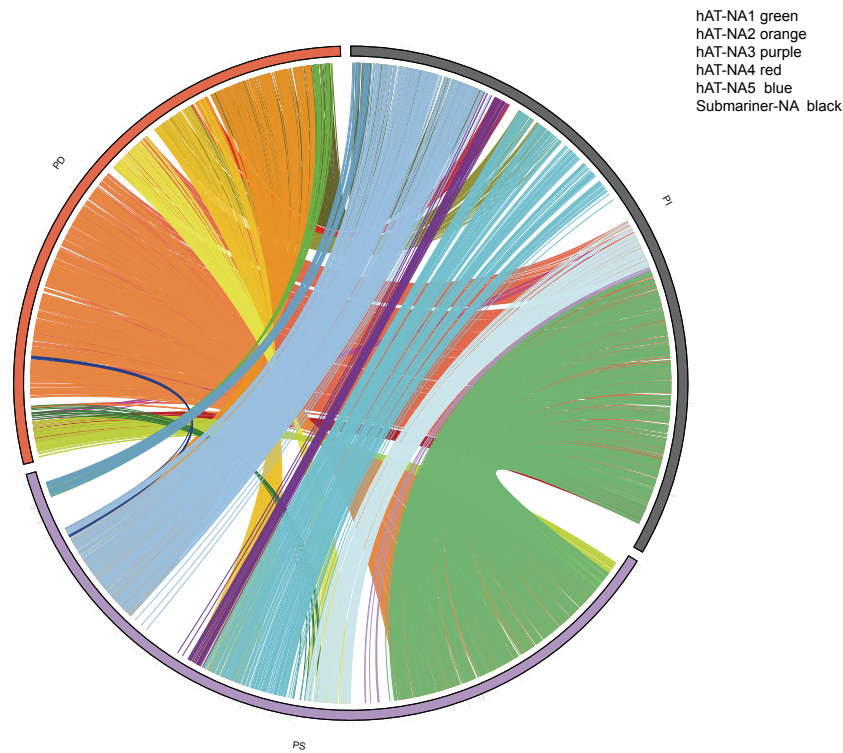

B

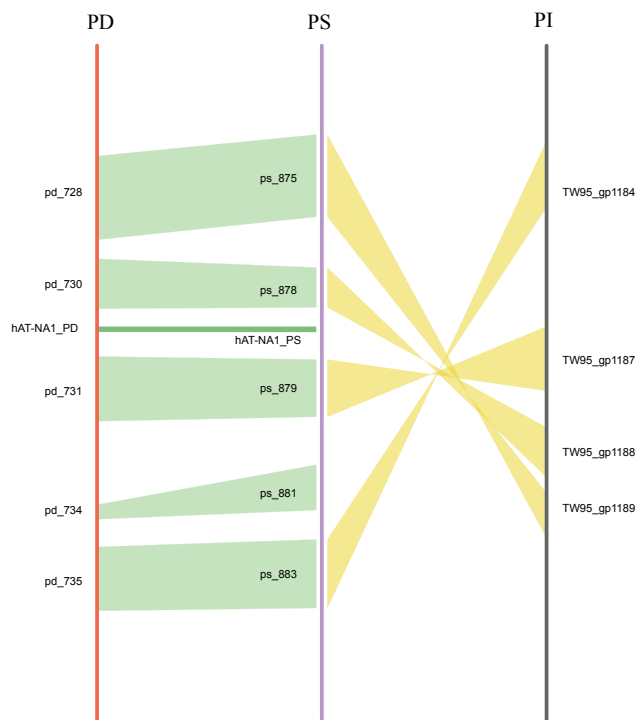

C

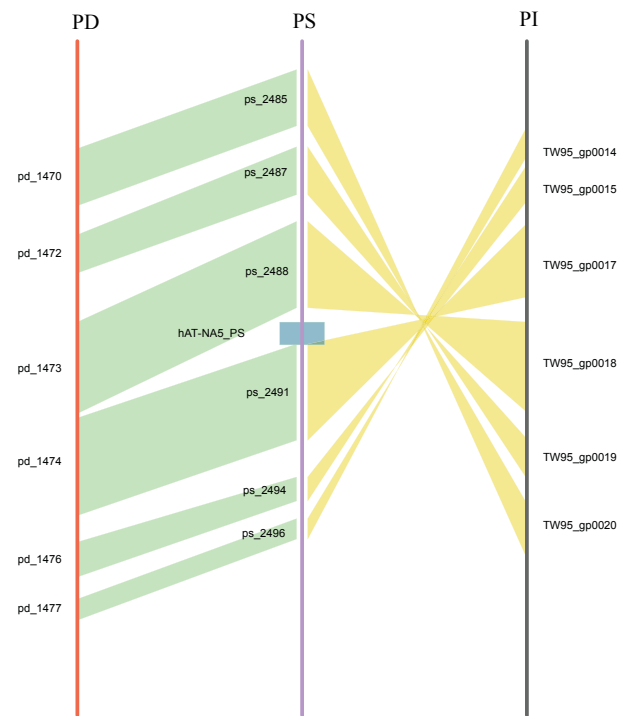

Supplement: Supplementary file 4 — Figure S2. Gene synteny and collinearity of three Pandoraviruses and distribution of six MITEs on its genomes. A Circle plot showing patterns of synteny and collinearity of three Pandoraviruses. MITEs on the genomes of three Pandoraviruses are shown using different colored lines. B One example of the present of one copy of MITEs in orthologous genomic site of P. salinus and P. dulcis but absent in that of P. inopinatum. C One example of the absence of one copy of MITEs orthologous genomic site of P. inopinatum and P. dulcis but present in that of P. salinus. (PDF 431 kb) [file 13100_2018_125_MOESM4_ESM.pdf]

# Additional file 5: Figure S3.

A

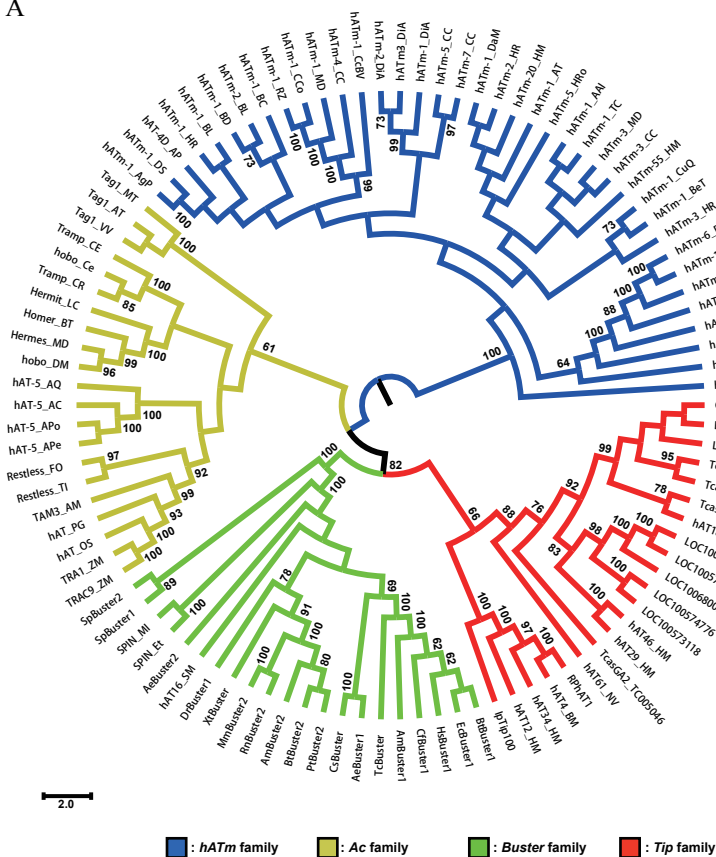

B

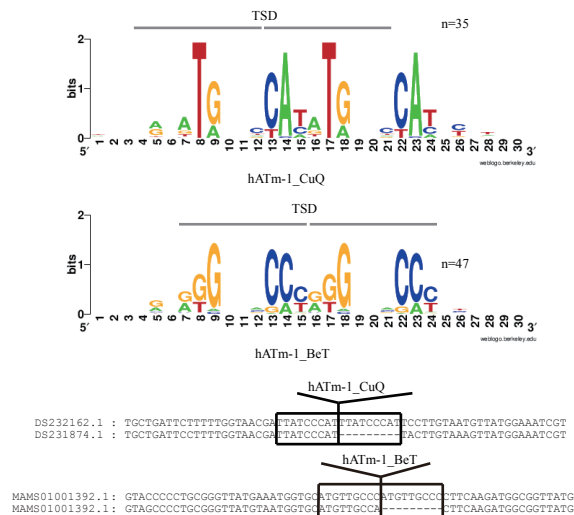

C

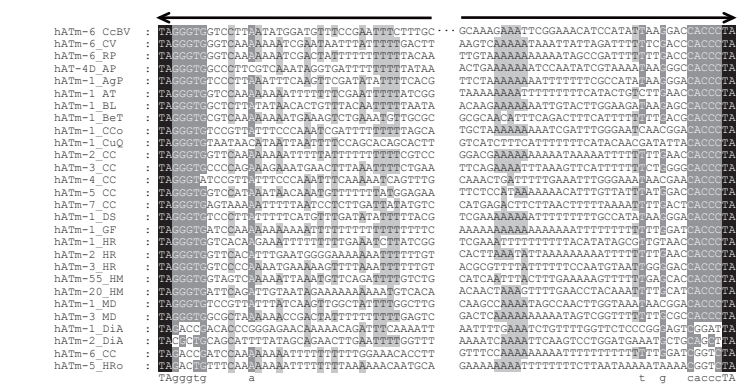

Supplement: Supplementary file 5 — Figure S3. Phylogeny of three known families (Buster, Ac and Tip) of the hAT transposons with the hATm family and characteristics of TSD of two members of the hATm family. A Phylogenetic analysis of hAT transposases, performed through Maximum Likelihood method. Numbers at nodes represent bootstrap support after 500 replicates. Four members (hAT5-AC, hAT5_AP, hAT5_APo and hAT5_AQ) of the Ac family were found in this study. The detail information of the hATm transposons is listed in Additional file 1: Table S2. B TSD of hATm-1_CuQ and hATm-1_BeT. The results of sequence logos and empty paralogous site analysis indicated that hATm-1_CuQ and hATm-1_BeT created a 9-bp TSD upon insertion. C Multiple alignments of the 5′ and 3′ terminal sequences of 28 hATm transposons identified in this study. Because hATm transposons are generally flanked by long TIRs (e.g. TIRs of hATm-6_RP are 417 bp), and only 40 bp of both termini of these transposons were shown arrows. (PDF 1651 kb) [file 13100_2018_125_MOESM5_ESM.pdf]

A

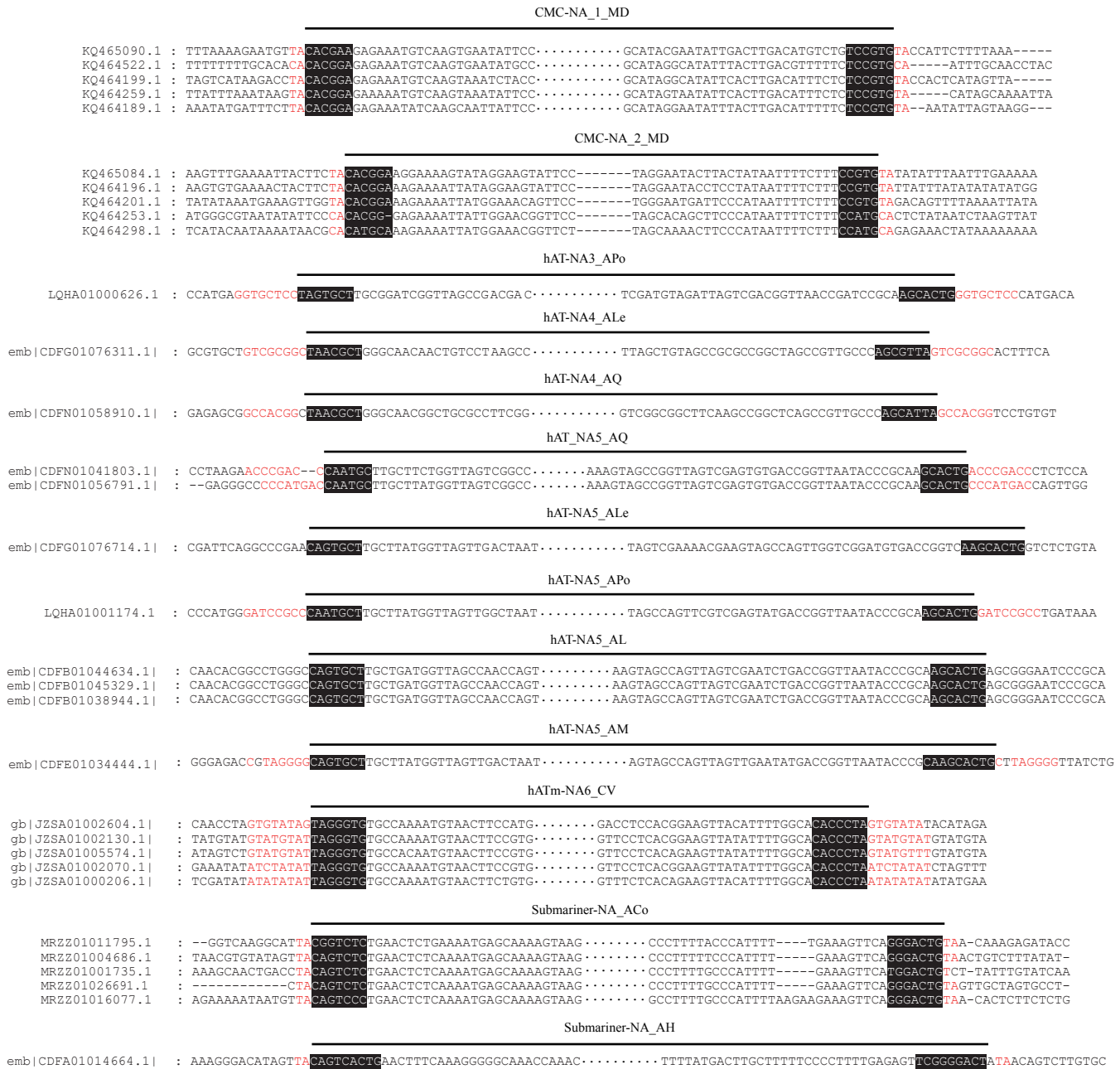

B

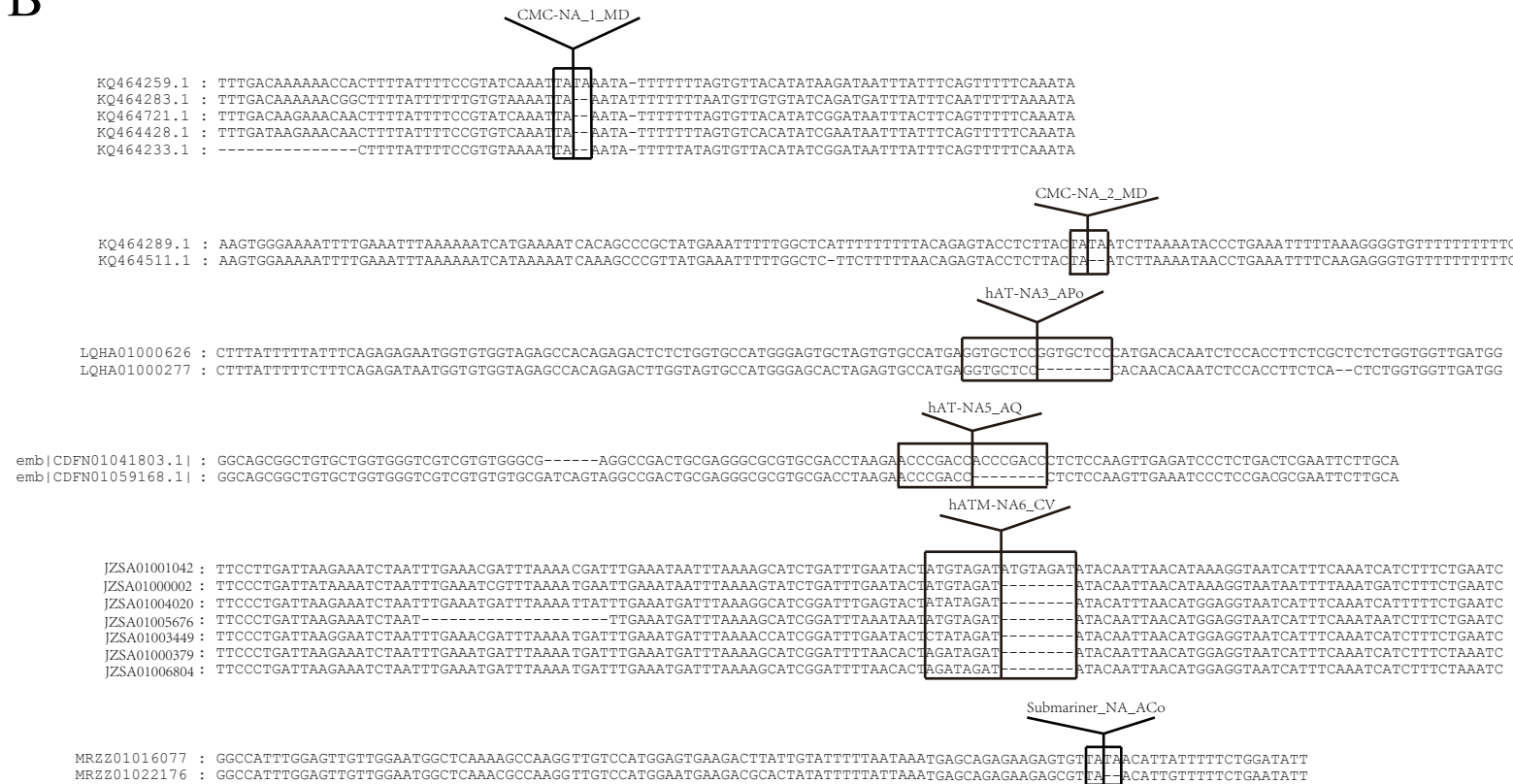

Supplement: Supplementary file 7 — Figure S4. Determination of the boundary and TSD of seven MITEs identified in viral hosts or species related to their hosts. A Multiple alignments of full-length copies as well as the flanking sequences of each MITE. TSD is shown using red color and the boundary is indicated usingi black shading. B Empty paralogous sites of five MITEs identified in viral cellular hosts. (PDF 2219 kb) [file 13100_2018_125_MOESM7_ESM.pdf]

Additional file 10: Figure S6.

A

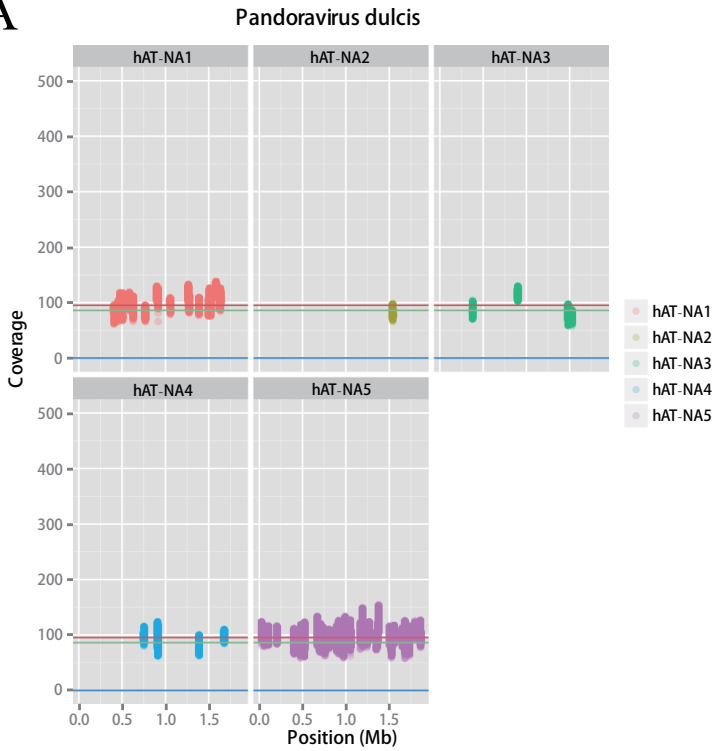

B

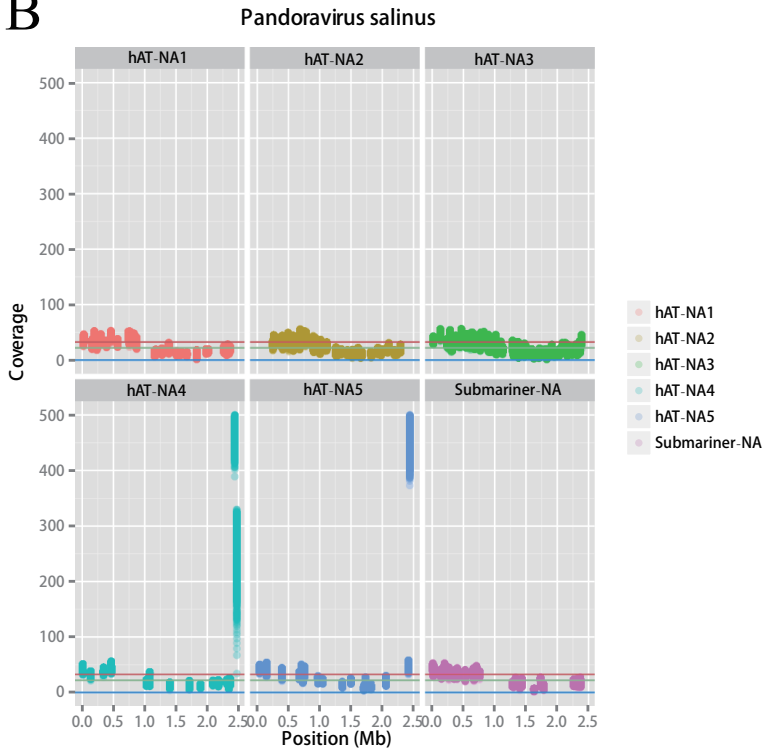

Supplement: Supplementary file 10 — Figure S6. Mapping MITEs on the P. salinus and P. dulcis genomes. The 2 kb fragments coverage centered on each MITE copy including hAT-NA1, hAT-NA2, hAT-NA3, hAT-NA4, hAT-NA5 and Submariner-NA for P. salinus (A) and hAT-NA1, hAT-NA2, hAT-NA3, hAT-NA4, hAT-NA5 for P. dulcis (B) The blue horizontal line corresponds to zero coverage, the red line is the mean coverage and the green line is the median. (PDF 376 kb) [file 13100_2018_125_MOESM10_ESM.pdf]
