# Supplementary material for: Unexpected invasion of miniature inverted-repeat transposable elements in viral genomes
Source: Mob DNA. 2018 Jun 18;9:19. doi: 10.1186/s13100-018-0125-4 (PMC6004678; doi:10.1186/s13100-018-0125-4)
Supplement: Supplementary file 6 — Table S3. Target site duplications of hATm transposons. (DOC 59 kb) [file 13100_2018_125_MOESM6_ESM.doc]

Additional file 6 : Table S3 Target site duplications of *hATm* transposons

| Species | TEs | Copies used | TSD |
| --- | --- | --- | --- |
| *Acyrthosiphon pisum* | *hAT-4D_AP^#^* | 1 | ATATATAT |
| *Aedes albopictus* | *hATm-1_AAl* | - | - |
| *Agrilus planipennis* | *hATm-1_AgP* | 1 | ATACTCAC |
| *Amyelois transitella* | *hATm-1_AT* | 2 | MTRSATAM |
| *Bactrocera cucurbitae* | *hATm-1_BC* | - | - |
| *B. latifrons* | *hATm-1_BL* | 9(4) | VTKDAYAB/VTRKRDCAT |
| *B. latifrons* | *hATm-2_BL* | - | - |
| *B. dorsalis* | *hATm-1_BD* | - | - |
| *Bemisia tabaci* | *hATm-1_BeT* | 78(47) | KSNNVSYN/NNVNNNVMN |
| *Cotesia congregata* | *hATm-1_CCo* | 1 | TTACGACAT |
| *C. congregata bracovirus* | *hATm-6_CcBV* | 1 | - |
| *C. sesamiae Kitale bracovirus* | *hATm-1_CsBV* | - | - |
| *C. vestalis* | *hATm-6_CV* | 10 | VYVNNBVN |
| *Culex quinquefasciatus* | *hATm-1_CuQ* | 47(35) | NTGBNYAN/NTRNNNYAH |
| *Cyphomyrmex costatus* | *hATm-1_CC* | - | - |
| *C. costatus* | *hATm-2_CC* | 1 | ATATATAT |
| *C. costatus* | *hATm-3_CC* | 2 | RTRMGYAT |
| *C. costatus* | *hATm-4_CC* | 12 | VWRHRBRB |
| *C. costatus* | *hATm-5_CC* | 4 | - |
| *C. costatus* | *hATm-6_CC* | 3 | WTRTAYAT |
| *C. costatus* | *hATm-7_CC* | 1 | ATATATAT |
| *Daphnia magna* | *hATm-1_DaM* | - | - |
| *Diachasma alloeum* | *hATm-1_ DiA* | 2 | WTMKACAS |
| *D. alloeum* | *hATm-2_ DiA* | 3 | ATRYVSAK |
| *D. alloeum* | *hATm-3_ DiA* | - | - |
| *Drosophila suzukii* | *hATm-1_DS* | 2 | ATAYATAT |
| *Glyptapanteles flavicoxis* | *hATm-1_GF* | 1 | ATATATAT |
| *Helobdella robusta* | *hATm-1_HR^#^* | 21 | DTRHNBAH |
| *H. robusta* | *hATm-2_HR^#^* | 11 | VYDBAYAB |
| *H. robusta* | *hATm-3_HR^#^* | 6 | ATATATAT |
| *H. robusta* | *hATm-5_HRo^#^* | 6 | MTRBATAY |
| *Hydra vulgaris* | *hATm-55_HM^#^* | 14 | DYNNNYAT |
| *H. vulgaris* | *hATm-20_HM^#^* | 42 | WWRTAYAY |
| *Microplitis demolitor* | *hATm-1_MD* | 2 | MTMWACAR |
| *M. demolitor* | *hATm-2_MD* | - | - |
| *M. demolitor* | *hATm-3_MD* | 1 | ATATATAT |
| *Rhagoletis zephyria* | *hATm-1_RZ* | - | - |
| *Trachymyrmex cornetzi* | *hATm-1_TC* | - | - |
| *Rhodnius prolixus* | *hATm-6_RP* | 20 | NTVNNBAN |

Note: - indicated that full-length copy or TSD of *hATm* transposons could not be determined in the corresponding species. N = A/T/C/G; W = A/T; D = A/T/G; V = G/A/C; M = A/C; R = A/G; Y = C/T; S = G/C; B = G/T/C; H = A/T/C; K = G/T. The numbers in parentheses represented members of *hATm*, which are integrated into 9-bp TSD. *^#^* showed that these *hATm* transposons were obtained from Repbase.
