# Supplementary material for: Unexpected invasion of miniature inverted-repeat transposable elements in viral genomes
Source: Mob DNA. 2018 Jun 18;9:19. doi: 10.1186/s13100-018-0125-4 (PMC6004678; doi:10.1186/s13100-018-0125-4)
Supplement: Supplementary file 8 — Table S4. Pairwise comparison of nucleotide sequence identity of seven MITEs involved in HTs between viruses and their cellular hosts or species closely related to their hosts. (DOC 68 kb) [file 13100_2018_125_MOESM8_ESM.doc]

Additional file 8: Table S4 Pairwise comparison of nucleotide sequence identity of seven MITEs involved in HTs between viruses and their cellular hosts or species closely related to their hosts

| ***CMC-NA_1*** | | | | | | | | | | | | | | | | | | |
| --- | --- | --- | --- | --- | --- | --- | --- | --- | --- | --- | --- | --- | --- | --- | --- | --- | --- | --- |
|  | | *Glypta fumiferanae* | *Microplitis demolitor* | |  | |  | | | |  | | |  | | |  | |
| *G. fumiferanae ichnovirus* | | 93.9 (164) | 96.9 (164) | |  | |  | | | |  | | |  | | |  | |
| ***CMC-NA_2*** | | | | | | | | | | | | | | | | | | |
|  | | *Cotesia congregata* | *C. vestalis* | | *M. demolitor* | | | *G. flavicoxis* | | | | *G. indiensis* | | | |  |  | |
| *C. congregata bracovirus* | | 96.6 (181) | 76.8 (220) | | 87.3 (245) | | | 86.2 (145) | | | | 73.0 (178) | | | |  |  | |
| *M. mediator bracovirus* | | 80.8 (178) | 71.0 (207) | | 79.8 (228) | | | 79.3 (145) | | | | 69.6 (178) | | | |  |  | |
| *G. flavicoxis bracovirus* | | 78.3 (162) | 73.3 (150) | | 81.7 (170) | | | 78.1 (142) | | | | 70.6 (160) | | | |  |  | |
| ***Submariner-NA*** | | | | | | | | | | | | | | | | | | |
|  | *Acanthamoeba comandoni* | | | *A. healyi* |  | | |  | | | |  |  | | | |  | |
| *Pandoravirus salinus* | 70.2 (242) | | | 72.3 (242) |  | | |  | | | |  |  | | | |  | |
| *P. inopinatum* | 63.7 (240) | | | 64.5 (240) |  | | |  | | | |  |  | | | |  | |
| ***hAT-NA3*** | | | | | | | | | | | | | | | | | | |
|  | *A. lugdunensis* | | | *A. polyphaga* |  |  | | | |  | | |  | | | |  | |
| *P. salinus* | 96.1 (103) | | | 90.4 (126) |  |  | | | |  | | |  | | | |  | |
| *P. inopinatum* | 96.1 (103) | | | 91.2 (126) |  |  | | | |  | | |  | | | |  | |
| *P. dulcis* | 92.2 (103) | | | 90.4 (126) |  |  | | | |  | | |  | | | |  | |
| ***hAT-NA4*** | | | | | | | | | | | | | | | | | | |
|  | *A. lenticulata* | | | *A. polyphaga* | *A. quina* |  | | | |  | | |  | | | |  | |
| *P. salinus* | 84.8 (304) | | | 75.4 (204) | 82.7 (261) |  | | | |  | | |  | | | |  | |
| *P. inopinatum* | 75.5 (295) | | | 66.6 (201) | 71.9 (257) |  | | | |  | | |  | | | |  | |
| *P. dulcis* | 83.6 (287) | | | 76.6 (184) | 82.1 (247) |  | | | |  | | |  | | | |  | |
| ***hAT-NA5*** | | | | | | | | | | | | | | | | | | |
|  | *A. mauritaniensis* | | | *A. lugdunensis* | *A. lenticulata* | *A. castellanii* | | | | *A. pearcei* | | | *A. polyphaga* | | | | *A. quina* | |
| *P. salinus* | 80.9 (346) | | | 81.0 (316) | 81.9 (299) | 78.3 (194) | | | | 78.3 (194) | | | 82.3 (346) | | | | 85.3 (335) | |
| *P. inopinatum* | 85.7 (413) | | | 87.8 (385) | 88.5 (367) | 81.6 (251) | | | | 81.6 (251) | | | 82.0 (413) | | | | 88.5 (402) | |
| *P. dulcis* | 86.7 (422) | | | 87.5 (392) | 88.0 (375) | 86.5 (260) | | | | 86.5 (260) | | | 85.3 (422) | | | | 90.9 (411) | |
| ***hATm-NA6*** | | | | | | | | | | | | | | | | | | |
|  | *C. vestalis* | | | *C. congregata* | *G. indiensis* | | | |  | | |  | | |  | | |  |
| *C. congregata bracovirus* | 88.1 (278) | | | 81.3 (279) | 81.9 (277) | | | |  | | |  | | |  | | |  |
| *C. vestalis bracovirus* | 79.8 (353) | | | 86.0 (352) | 87.5 (354) | | | |  | | |  | | |  | | |  |
| *C. sesamiae Kitale bracovirus* | 84.7 (354) | | | 78.4 (353) | 78.1 (353) | | | |  | | |  | | |  | | |  |
| *C. sesamiae Mombasa bracovirus* | 85.0 (354) | | | 78.4 (353) | 78.1 (353) | | | |  | | |  | | |  | | |  |
| *C. plutellae polydnavirus* | 78.4 (339) | | | 73.2 (337) | 72.1 (337) | | | |  | | |  | | |  | | |  |

Note: Numbers in parentheses represent the overlap nucleotides of one MITE between viruses and their cellular hosts or species closely related to their hosts.
