# Supplementary material for: Unexpected invasion of miniature inverted-repeat transposable elements in viral genomes
Source: Mob DNA. 2018 Jun 18;9:19. doi: 10.1186/s13100-018-0125-4 (PMC6004678; doi:10.1186/s13100-018-0125-4)
Supplement: Supplementary file 9 — Figure S5. Multiple alignments of seven MITEs involved in HTs between viruses and its cellular hosts or species related to their hosts. (PDF 5206 kb) [file 13100_2018_125_MOESM9_ESM.pdf]

Additional file 9: Figure S5.

A

```
Submariner-NA_PS    CAGTCGCCGAACTCTCAAAGGGGGCAAAAGAAAGTCATAAAAAGTCAAAGGGACGTCCCAAAAGTGTCTACAG-CCTGTTGTTTTGCCTGCTTGAAAAT-
Submariner-NA_PI    CAGTCGTCGAACTCTCAAAGGGGGCAAAATAAAGTCATGAAAAGTCAAAGGGACGTCCCAAAAGCGTCCACAG-CCCGTTGTTTTACCTGCTTGGGGATG
Submariner_NA_ACo   CAGTCTCTGAACTCTCAAAATGAGCAAAAGTAAGTCATAATAAGTCAACGGTACGCACTGAACGTGTCGACGT-GGTGCTGTTTTTGCTGCTTACACTT-
Submariner_NA_AH    CAGTCACTGAACTTTCAAAGGGGGCAAACCAAACTCACAGACAGGAAAAAAATCGTCACAAGCGTGTCTACAGCCCCCTTGTTTTCTCTGCTTACGTTC-

Submariner-NA_PS    TGTAGACATAT--GGAGACGAGACATGTCTGGTGTCGGCACTTTTGCGCATT-CCCAAGCAGGCAAAACAACAGGCTGTAGACACTTTTGGGACGCTTTG
Submariner-NA_PI    CGCAAAAATGC--CGACATCAGACATGTCTCGCCCCCGTATGTCT--ACATT-TTTAAGCAGACAAAACAACGGGCTGTGGACGCTTTTGGGACGTCCCT
Submariner_NA_ACo   TGCAGACAATCTGCAATTTGCGACATGTCTCTTGTCTGCAGTT-TGCACATCGCTCAAGCAGTGGGAACAAAGGAATGTAGACATGACAGAGACGCCTGC
Submariner_NA_AH    TGTAGACAAATT-TGTTGTGGGACATGACTGTTCTCGGCAGTTGAGTTTTGC-CTCAAGCAGGAAAAACTTGCTGTTGTAGACACTCTTTGTACACTCCA

Submariner-NA_PS    TTGACTTTTTATG--ACTTTCTTTTGCCCCCTTTGACAGTTCGGCGACTG
Submariner-NA_PI    TTGACTTTTCATG--ACTTTATTTTGCCCCCTTTGAGAGTTCGACGACTG
Submariner_NA_ACo   TTGCCTTCATTTGGCGCTCCCTTTTGCCCATTTTGAAAGTTCAGGGACTG
Submariner_NA_AH    TTGACTTTTTATG--ACTTGCTTTTTCCCCTTTTGAGAGTTCGGGGACT-
```

B

```
CMC-NA_1_GfIV   CACGGAGAGAAATTTCAAGTAAATATTCCTATGCAGCATAGTAATGGTTACATTACTCTATAGTTTGTATAA----GCGGTCACATAAGAGCGGCGTGAC
CMC-NA_1_GF     CACGGAGAGAAATATCAAGTAAATGTTCCTACGTAGCATAGTAACGGTTGCATTCCTCTATGGTTTGTGTCACGCCGCTCTTATGTGACTGC----TTAT
CMC-NA_1_MD     CACGGAGAGAAATGTCAAGTAAATATGCCTATGCAGCATAGTAATGGTTACATTACTCTATAGTTTGTGTCACGCCGCTCTTATGTGACTGC----TTAT

CMC-NA_1_GfIV   ACAAAC--TATAGAGTAATGTAACCGTTACTATGCTGCATAGGAATATTTACTTGATATTTCTCTCCGTG
CMC-NA_1_GF     ACCAACTATATAGAGTAATGTAACCATTACTATGCTGCATAGGAGTATTTACTTGAAATTTTTCTCCGTG
CMC-NA_1_MD     ACAAAC--TATAGAGTAATGTAACCATTACTATGCTGCATAGGAATATTTACTTGACATTTCTCTCCGTG
```

C

```
CMC-NA_2_CcBV   CACGGAAAGAAAAATATGGGAACTATTCCCATAATTTTATGGGAACAGTTCCCAGGC-----------------------ATTATGGGAACCATTCCTAT
CMC-NA_2_MmBV   ------------ATTATGGGAATGCTTCCCATAATGTTATGGGAATAGTACCTATACTACTATAGGGATGGTTCCCATATATTATGGGAACCATCCCCAT
CMC-NA_2_GfBV   --------------------------------------------------------------------------------ATTATAGGAACCGATACTAT
CMC-NA_2_CV     CACGGAAAGAAAAATA-GGGAACGGTTCCCATAATTTCATGAAATTTTTTCCTTTTC----------------------TATTATAGGAACTACGACTAT
CMC-NA_2_GI     ------------------------ATTCCCATATT---------------------------------------------ATTATAAGAATTTTTCCTAT
CMC-NA_2_GF     ----------------------------------------------------------------------------------------AATCATTCCTAT
CMC-NA_2_MD     CACGGAAAGAAAATTATAGGAACTGTTCCTATA-TATTATGGGAATGGTTCCCATAC----------------------TATTATAGGAACTATTACCAT
CMC-NA_2_CC     --------------------------------------------------------------------------------------------ATTCCTAT

CMC-NA_2_CcBV   AATATTATAGGAATAATTCCTATAGCATTATAGGTACCATTCCCATAATATTATGGGATTAGTTCCCATAATAATGTAGGAATAGTTTCTATAATAGTAT
CMC-NA_2_MmBV   AATAGTATGAGAATAGTTCCCATACCATTATGGGAATGGTTCCCATAATATTATGGGATTGGTTCCCATATTGGTATAGGAATGGTTCCCATATTGGTAT
CMC-NA_2_GfBV   AATGTTATAGGAATAGTTCCCATA--ATTATAGGAACAGTTCTTAWWA-ATTATAGGAACAGTTCTTATAA-ATTATAGGAACAGTTCCAATATCATTAT
CMC-NA_2_CV     AA-ATTATAGGAACTGTTCCTATA-------------------------CCTATGAGAATGCTACTCATGATATTATAGGAATGGCTCCCATAGT-TTAT
CMC-NA_2_GI     ACTATGATAGAAACCATTCTCATACTATTATGGAAGTT-----------GTTATAGAAATAGTTTCCATACTATTATGGAAGTTGTTCCCATAGTAATAT
CMC-NA_2_GF     AGTAATACAGGAATAATTCCTATA-------------------------ATTATGGGAGTGGTTCCCATAAT--TATAGGAATGGTTCCTATACTATTGT
CMC-NA_2_MD     AA-ATTATAGGAACAGTTCCTATA--ATTATAGGAATCATTCCCATA--ATTATAGGAATGGTTCCCATAATG-TATAGGAACGATTCCTATAATATTAT
CMC-NA_2_CC     AATAGTATAGGAATGATTCCTATAGCATTATGGGAACCATTCCCATAATATTATGGGATTAGTTCCCATAATGATATAGGAATAGTTTCTATAATAGTAT

CMC-NA_2_CcBV   AGGAATTATTCCTATAGCATTATGGGAACCATTCCCATAATGGTATAGGAATAGTTCCCATAATTTTCTTTCCCTG
CMC-NA_2_MmBV   AGGAACTATTCCTATAATATTATGGGAACTATTCCCATAATGTTATGGGAACCATCCCTATAATATCATA------
CMC-NA_2_GfBV   GAGAACTATTCCCATA--ATTATGGGAACCATTCCTATAAT--TATAGGAATTATTCCCATA--------------
CMC-NA_2_CV     AGGAATGGTTCCTATACCTTAATGGGAACCATTCCCATAGC-GTATAGGAAAAATTCTTAAAATTTTCTCTC----
CMC-NA_2_GI     AGGAATTTTTCTTATACTATTATGGGAACCATTCTGATATCAGTATAGGAAGAATTCC------------------
CMC-NA_2_GF     AGGAATGATTACCATA--ATTATAGGAATAATTCCTATAAT--TATAGGAATAATTCCCATAAT------------
CMC-NA_2_MD     AGGAATGATTCCTATATCATTATGGGAACCATTCCCATAAT-ATATAGGAATAGTTCCCATAATTTTCTTTCCGTG
CMC-NA_2_CC     AGGAATTATTCCTATAGCATTATGGGAACCATTCCCATAATGGTATAGGAATAGTTCCCATAATTTTCTTTCC---
```

D

```
hAT-NA3_PS    CAGTGCTTGCGGATCGGTTAACCGACGACTAAAATCCAGGATTTTAGAGGGATTTTTTGATTTATATGATTTTTGATTGGACAATTTGGTGTGGATTAGC
hAT-NA3_PI    CAGTGCTTGCGGATCGGTTAACCGACGACTAAAATCCAGGATTTTAGGGGGATTTTTTGATTTATATGATTTATGATTGGACAATTTGGTGTGGATTAGT
hAT-NA3_PD    -AGCGCTTGCGGATCGGTTAACCGGCGACTAAAATCCAGGATTTTAGACGGATTATTTGATTTATATGATTTATGATTGGACAATTCTGTGTGGATTAGT
hAT-NA3_APo   -AGTGCTTGCGGATCGGTTAGCCGACGACTAAAATCCAGGATTTTGGACGGATTGTTTGATTTATAAGATTTATGATTGGGCAATTCGATGTAGATTAGT
hAT-NA3_AL    ------------ATCGGTTAGCCGACGACTAAAATGCAGGATTT-----------TTTGATTTATATGATTTTTGATTGGACAATTTGGTGTAGATTAGT

hAT-NA3_PS    CGACGGCTAACCGATCCGCAAGCACTG
hAT-NA3_PI    CGACGGCTAACCGATCCGCAAGCACTG
hAT-NA3_PD    CCACGGCTAACCGATCCGCAAGCACTG
hAT-NA3_APo   CGACGGTTAACCGATCCGCAAGCACTG
hAT-NA3_AL    CGACGGCTAACCGATCCGCAAGCACT-
```

E

```
                     10        20        30        40        50        60        70        80        90       100
             ....|....|....|....|....|....|....|....|....|....|....|....|....|....|....|....|....|....|....|....|
hAT-NA4_PI   TAGCGCCGGGCAATGGCTGCGCCCAAGCCGGCCAACCGACATGATCGAGCCAACCGATTGAAGCCGACCACAGGCCACC---GACAGAAAGGAGTGCCAG
hAT-NA4_PS   TAACGCTGGGCAACGGCTGGGCCTAAGCCGGCCAGCCGGCACGCTCGAGCCAACCGGCTCAAGCCGGCCACAAGCCGCC---GACAAAAAGGAGTGCGAG
hAT-NA4_PD   TAACGCTGGGCAACGGTTGCGCCTAAGCCGGCTAACCGACATCCTCTAGCCAACCGGATGAAGCCGGATACAAGCCGTC---GACAAAAATGA-------
hAT-NA4_APo  TAACGCTGGGCAACGTCTGCGTCTAGGCCGGATAGCCAAT-TTCTCGAGCCAACGGGCAGAAGCCGGCTTGAAGCCTTTCCGTACACATAGGAACACTTG
hAT-NA4_AQ   TAACGCTGGGCAACGGCTGCGCCT---TCGGCTAGCCGACACTCTCTAGCCAACCGGCTGGAGCCGGCTGAAAGCCGTC---GACAAAAAAGA------G
hAT-NA4_ALe  TAACGCTGGGCAACAACTGT-CCTAAGCCGGTTAA-CGATGTTCTCAAGCCAACCGGCTGAAGCTGGCTACAAGCTGTC---GATAAAAAGGG--GCGAG

                    110       120       130       140       150       160       170       180       190       200
             ....|....|....|....|....|....|....|....|....|....|....|....|....|....|....|....|....|....|....|....|
hAT-NA4_PI   TACGAATGAAATA--TGCACAATGTAAGCGCGACAGTGTCACGG------ATGCG---------------------------------------------
hAT-NA4_PS   CGCGAACAAAATG--CGCACAATTTGAGCACGACGATATTGCGAGC------GCG---------------------------------------------
hAT-NA4_PD   ----------------GCACAATTTGAGCACGATGCTGCCGCGAGCGCGAATGCG---------------------------------------------
hAT-NA4_APo  TGCGATTATTATGGTTGCACAATTTGAGCACAATGTCATTCCGCATGCGAATGCG---------------------------------------------
hAT-NA4_AQ   CGCGAGTAAAATG--CGCACGATTTGAGCACAATGTTGTTGCGAGTGGGAATGCGCACAGTGAGAACGGCTTGCAGAAAACTGGCCTTTCGAGGCGTCTG
hAT-NA4_ALe  TGCGACTAAAATG--CGCACAATTTGAGCACGATGCTATCGCGAGTGCGAATGCG---------------------------------------------

                    210       220       230       240       250       260       270       280       290       300
             ....|....|....|....|....|....|....|....|....|....|....|....|....|....|....|....|....|....|....|....|
hAT-NA4_PI   ------ACCAAGCCGACCGCGAA-----------------------------------------------------------------------------
hAT-NA4_PS   ------ACCAAGCCGACCGCGAA-----------------------------------------------------------------------------
hAT-NA4_PD   ------ACTAAGCCGACCGCGA------------------------------------------------------------------------------
hAT-NA4_APo  ------AATAAGCCGAGCGCGAA-----------------------------------------------------------------------------
hAT-NA4_AQ   GAGTTAACTTGGCGGACAACAGATTGAAATCTCCCAATTTTTGATGGGGTAAACGTGCTTTGGTCCTCAAACATTTTTTAGGATTTTTTGGCGTAGTGCC
hAT-NA4_ALe  ------ACTCAGCCGA-CGCGAA-----------------------------------------------------------------------------

                    310       320       330       340       350       360       370       380       390       400
             ....|....|....|....|....|....|....|....|....|....|....|....|....|....|....|....|....|....|....|....|
hAT-NA4_PI   ------------------------------CGCGAG--------------TTTACCGTTCACGATTTGTGCAC-AACATTACTAAATTCGTTCGCACCCG
hAT-NA4_PS   ------------------------------TGCGAGTTC-----------CTTGTTCTTCGCAATTTGTGCAC-AATGTTTCTACATTCATTCGCGCTCG
hAT-NA4_PD   ----------------------------------GTTTC-----------CTTATTCTTCACAATTTGGGCACAAATTTTTATGCATTGGTTCGCACTCG
hAT-NA4_APo  ------------------------------TGCGAGTCC-----------CTTATTCTTCAGAATTTGTGCGC-AATGTTTCTACA--------------
hAT-NA4_AQ   CGGCCTGAGGACCGTGGCGTTCGCACTGTGTGCGATTACTACGACCAGGGCTCGTTCTTCACAATTTGTGCAC-AATTTTACTTCATTCGCTCACACTCG
hAT-NA4_ALe  ------------------------------TGCGACTTA-----------ATTGTTCTTCTCAATTTGTGCAC-ATTGTTTCTACATGCATTCGCACTCG

                    410       420       430       440       450       460       470       480       490
             ....|....|....|....|....|....|....|....|....|....|....|....|....|....|....|....|....|....|..
hAT-NA4_PI   CGCTTC-TTTTTATCGACGGCTTGTAGCGGGCTTCAGCCTGTTGGCTCGACCCTTGGCCGC------GCCGGCTAGCCGTTGCCCAGCCTT-
hAT-NA4_PS   CGCTCC-TTTTTGTCGACGGCTTGTAGCCGGCTCCAGCCGGTTGGCTCGACCCTTGGCCGTAGCCG-GCCGGCTAGCCGTTGCCCAGCATTA
hAT-NA4_PD   CACCCC-TTTTTGGCGACGGCTTGTAGCCGGCTTCAGCCGGTTGGCTTGCCCCTTAGCCGAAGCCGAGCCGGCTAGCCGTTGCCCAGCGTTA
hAT-NA4_APo  --------------------------------------------------------------------------------------------
hAT-NA4_AQ   CACTTC-TTTTTGTCGGCGGCTTCAAGCCGGCTC----------------------------------------AGCCGTTGCCCAGCATTA
hAT-NA4_ALe  CACTTCTTTTTTGTCGGCGGCTCGTGGCAGGATTCAGCCGGTTGGCTCGACCCTTAGCTGTAGCCGCGCCGGCTAGCCGTTGCCCAGCGTTA
```

F

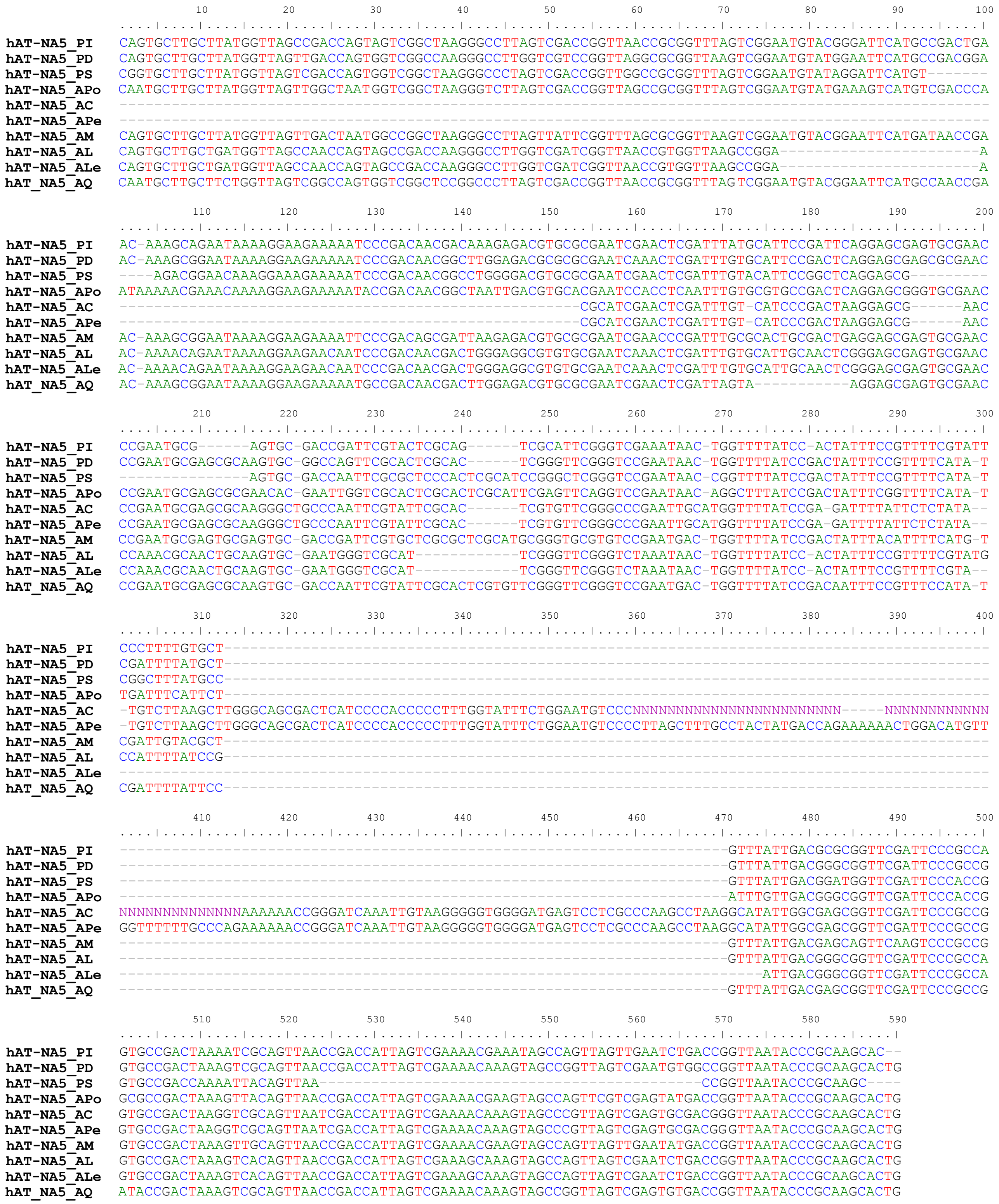
hAT-NA5_PI
hAT-NA5_PD
hAT-NA5_PS
hAT-NA5_APo
hAT-NA5_AC
hAT-NA5_APe
hAT-NA5_AM
hAT-NA5_AL
hAT-NA5_ALe
hAT_NA5_AQ

G

```
                        10        20        30        40        50        60        70        80        90       100
                ....|....|....|....|....|....|....|....|....|....|....|....|....|....|....|....|....|....|....|....|
hATm-NA6_CvBV   TAGGATGTGCCAAAATGTAACTTCCTTGATGGGCCTTTTAAAATTGGCATTTTGAGTTCCGCTTTTAACAGGAATTGTGTTTGGAAATTTCCTGACATAT
hATm-NA6_CpPV   TAGAGTTCGCCAAAATTCAATTTCCGTGAGAAACCTTTTAAAATTGGAATTTTGAGTTCTGCGTTTAACAGCAGCTGCGTTAGGGTGTCTTTCGACATCT
hATm-NA6_CsKBV  TAGGGTGCGCCAAAATGTAATTTCCGTGAAGAACCTTTTAAAATTGGAATTTTGAATTTCGCTTTTAACAGCAGCTGTGTTTAGGCATTTCTTGAAATAT
hATm-NA6_CsMBV  TAGGGTGCGCCAAAATGTAATTTCCGTGAAGAACCTTTTAAAATTGGAATTTTGAATTCCGCTTTTAACAGCAGCTGTGTTTAGGCATTTCCTGAAATAT
hATm-NA6_CcBV   TAGGGTGTGCCAAAATTCAATTTCCATGAGGAACCTTTAAAAATTGGAATTTTGAGTTTCGCTTTTAACAGGAGCTGTGTTTGGGCATTTCTTGAGATAT
hATm-NA6_GI     TAGGGTGTGCCAAAATGTAACTTCCTTGATGGACCTCTTAAAATTGTAATTTTGAGTTCCGCTTTTAACAGGAGTTGTGTCTGGGCATTTCCTAACATAT
hATm-NA6_CC     TAGGGCGTGCCAAAATGTATCTTCCTTGATGGACCTTTTAAAATTGGAATTTTGAGTTCCACTTTTAACAGGAGTTACGTTTGGGCATTTCCTGTCATAT
hATm-NA6_CV     TAGGGTGTGCCAAAATGTAACTTCCGTGAGGAACCTTTTAAAATTGGAATTTTGAGTTCCGCTTTTAACAGGGGCTGTGTTTGGGCATTTCCTGAGATAT

                       110       120       130       140       150       160       170       180       190       200
                ....|....|....|....|....|....|....|....|....|....|....|....|....|....|....|....|....|....|....|
hATm-NA6_CvBV   TTTGAACGTTAAGGATTTATTTGATTTTCATAGGATTTTTTACCAGGAGTTTTGTTTAGGCATTTTTGCTGGAATTTCAAAATTTCA--AACTTTGGCC-
hATm-NA6_CpPV   TTTGGGTTCAAACGATTTATTTGATTTTCAAAGAA-TTTTTAACGATGGCT----TTAGGCAC------CCGAATACGGAAATTTTAGCGGAA-TGCCC-
hATm-NA6_CsKBV  TTCGGGGTAAGAGGATTTATTTGATTTTCACAGAA-TTTTCAACAGGAGATTCAATTTGGCACTTCCAGCCAAATTTTGAATTTTCAGCGGGAATGCCC-
hATm-NA6_CsMBV  TTCGGGGTAAGAGGATTTATTTGATTTTCACAGAA-TTTTCAACAGGAGATTCAATTTGGCACTTCCAGCCAAATTTTGAATTTTCTGCGGGAATGCCC-
hATm-NA6_CcBV   TTTG---------------------------------------------------------------------------AAATTTCAGCGGAATTTCCCG
hATm-NA6_GI     TTTAAGCCTAACGAATTTATTAGATTTTCAGAAAATTTTTTAACAGGAGTTTTGATTAGGCATTTTTGCTGAAATTTCAAAATTTCA--AAATTTGGCC-
hATm-NA6_CC     TTTGAGCGTAAAGGATTTATTTGATTTTTATAGAATTTTTTAACAGGAGTTTTGTTCGGCTAAATTT--TGAAATTTTTAAATTCCAGCAAAAATGGCC-
hATm-NA6_CV     TTTGGGGTGAGACGATAAATTTGATTTTCATAGAATTTTTTAACAGAAGCTTAGTTTGGGCATTTCCGCTGAAAATTCAAAATTTGGCCGGAAGTGCCC-

                       210       220       230       240       250       260       270       280       290       300
                ....|....|....|....|....|....|....|....|....|....|....|....|....|....|....|....|....|....|....|
hATm-NA6_CvBV   --GAACAAAACTCCTGTTAAAAAATTCTTTAAAAATTAAATAAATCCTTAACGTTTAAAATATGTCAGGAAATTCCCAAACACAATTCCTGTTAAAAGCA
hATm-NA6_CpPV   --AAACAAAACTCTTGTT-CAAAATTCTATAAAAATCAATTGAATCGTCTCAACCCAAAATATCTTAGGAAATGCCCTAACATAGTTTCTGTTAAAAGCA
hATm-NA6_CsKBV  --AAACTAAGCTCCTGTTAAAAAATTCTATGAAAATCAAATTTATCGTCTCACCTCAAAATATCTCAGGAAATGCCCAAACACATCCTCTGTTAATAGCG
hATm-NA6_CsMBV  --AAACTAAGCTCCTGTTAAAAAATTCTATGAAAATCAAATTTATCGTCTCACCTCAAAATAACTCAGGAAATGCCCAAACACATCCTCTGTTAATAGCG
hATm-NA6_CcBV   GAAAGTAAAGCTCCTGTTAAAAAATTCTATGAAAACCAAATAAATCATTTTCCTCCAAAATATCTCAGAAAATGCCCAA--ACAGCTCCTGTTAAAAGCA
hATm-NA6_GI     --AAACAAAACTCTTGTTAAAAAATACTATGAAAATATAATAAATCCCTTACGCTCAAAATATGTCAGGAAATGCCCAGACACAACTCCTGTTAAAAGTG
hATm-NA6_CC     --GAACGAAACTCCTGATAAAAATTTCTGTAAAAATCAAATAAATCCTTTACGCCCAAAATTTGTCAGGAAATGCCCAACCACAACTCCTGTTAAAAGCG
hATm-NA6_CV     --AATTGAATCTCCTGTT-AAAAATTCTATGAAAATCAAATAAATCCTCTCACCCCGAAATATCTCAGGAAATGCCTAAACACAGCTGCTGTTAAAAGCG

                       310       320       330       340       350
                ....|....|....|....|....|....|....|....|....|....|....|....
hATm-NA6_CvBV   GGACTCAAAATGCCAATTTTAAAAGGTCCATCAAGGAAGTTACATTTTGGCACACCCTA
hATm-NA6_CpPV   GAACTCAAAATTCAAATTTTAAAAGGTTTCTCACGGAAATTGAATTTTGGCACAC----
hATm-NA6_CsKBV  GAACTCAAAATTTCAGTTTTAAAAGGTTCTTCACGGAAGTTACATTTTGGCACACCCTA
hATm-NA6_CsMBV  GAACTCAAAATTTCAGTTTTAAAAGGTTCTTCACGGAAGTTACATTTTGGCACACCCTA
hATm-NA6_CcBV   GAACTCAAAATTCCAATTTTAAAAGGTTCCTCACGGAAATTGAATTTTGGCAAACCCTA
hATm-NA6_GI     GAACTCAAAACTCCAATTTTAAGAGGTCCATCAAGGAAGTTACATTTTGGCACACCCTA
hATm-NA6_CC     GAACTCGAAATTTCAATTTTAAGAGGTCCATCAAGGAAGTTACATTTTGGCACACCCTA
hATm-NA6_CV     GAACTCAAAATTTTAATTTTAAAAGGTTCCTCACGGAAGTTACATTTTGGCACACCCTA
```
