# Supplementary material for: Unexpected invasion of miniature inverted-repeat transposable elements in viral genomes
Source: Mob DNA. 2018 Jun 18;9:19. doi: 10.1186/s13100-018-0125-4 (PMC6004678; doi:10.1186/s13100-018-0125-4)
Supplement: Supplementary file 11 — Figure S7. Phylogenies of full-length copies of five MITEs involved in HTs between viruses and their hosts. (PDF 482 kb) [file 13100_2018_125_MOESM11_ESM.pdf]

Additional file 11: Figure S7.

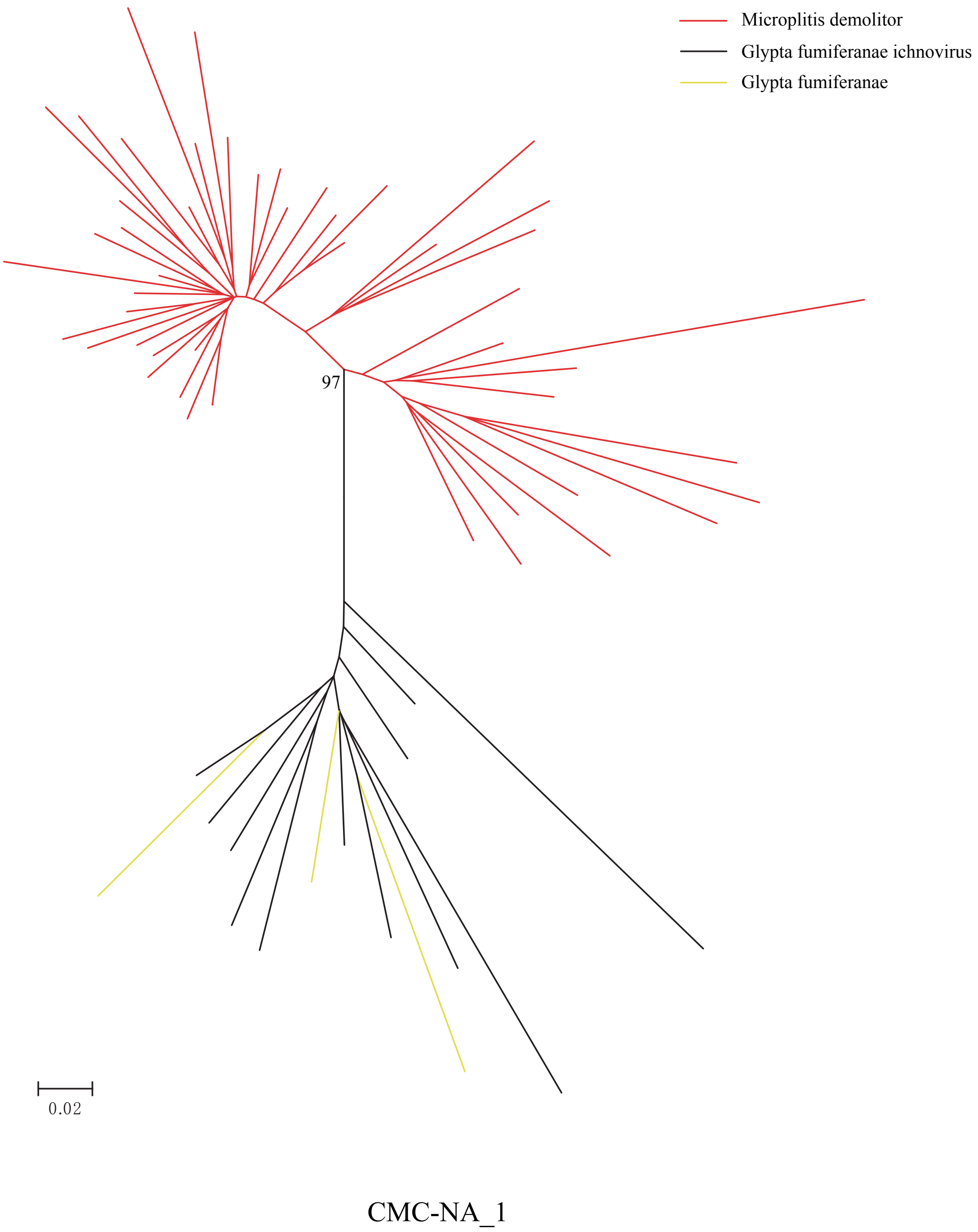

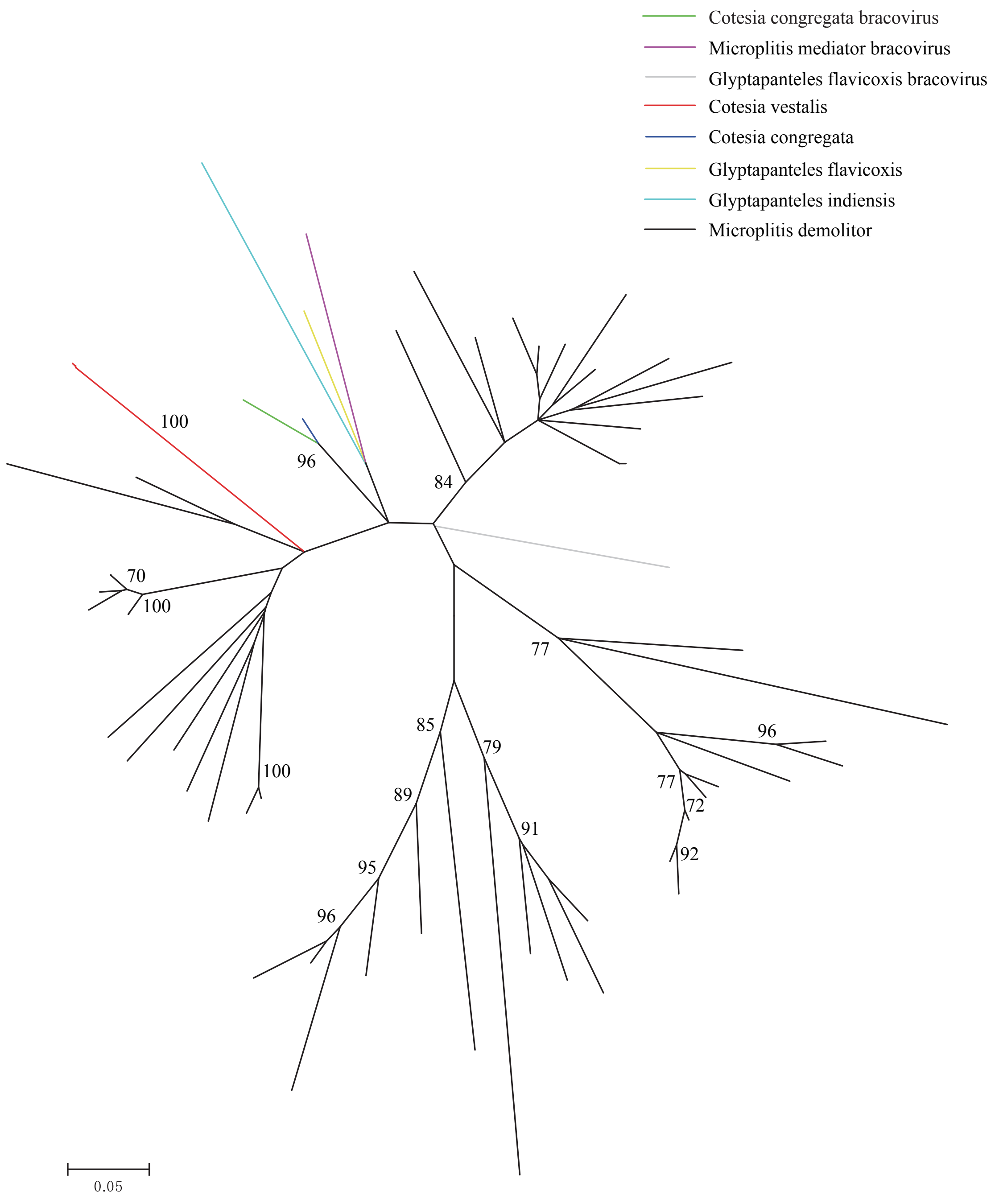
Cotesia congregata bracovirus
Microplitis mediator bracovirus
Glyptapanteles flavicoxis bracovirus
Cotesia vestalis
Cotesia congregata
Glyptapanteles flavicoxis
Glyptapanteles indiensis
Microplitis demolitor
100
96
84
70
100
77
100
85
79
96
89
77
72
91
95
92
96
0.05

CMC-NA_2

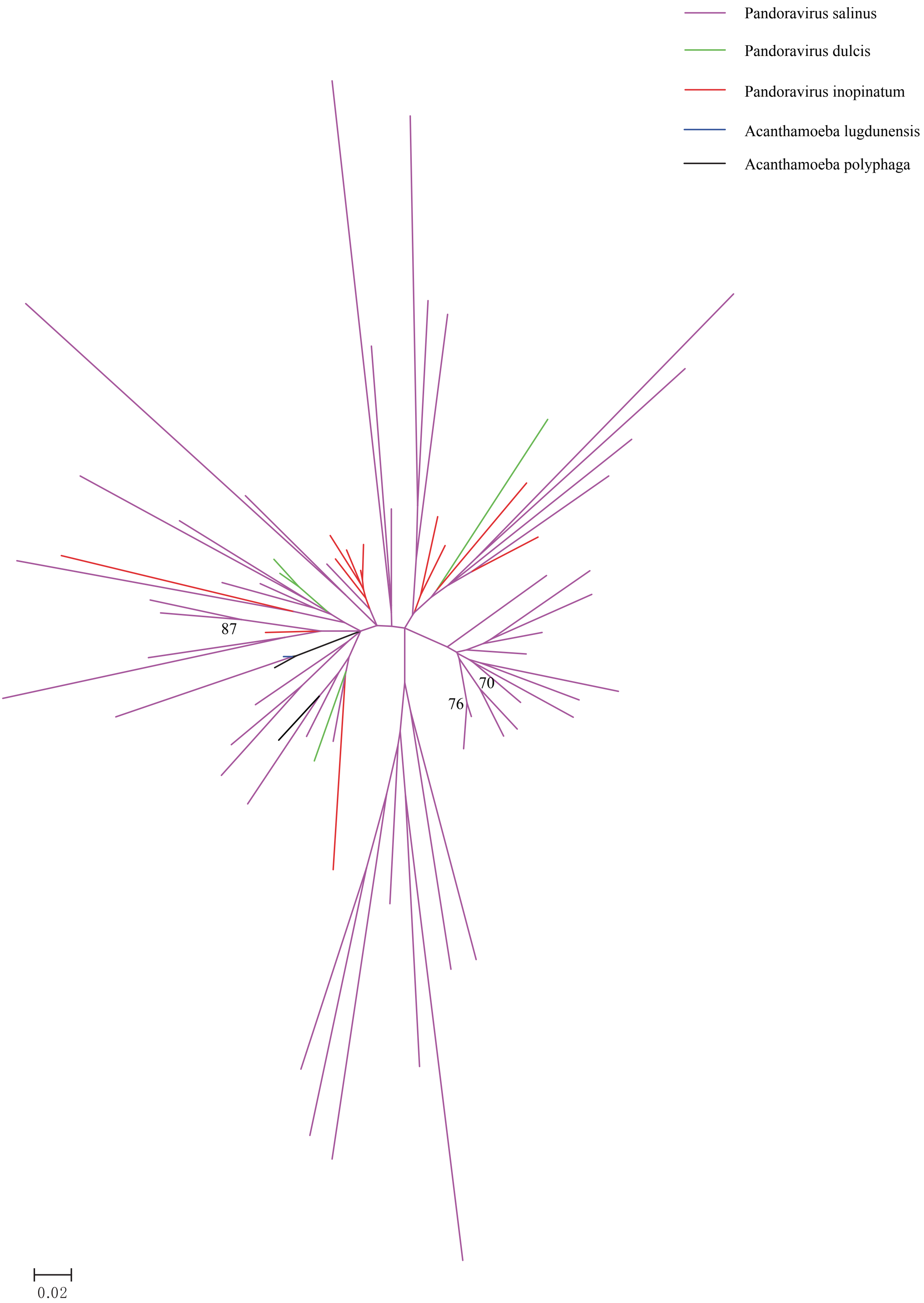

Pandoravirus salinus
Pandoravirus dulcis
Pandoravirus inopinatum
Acanthamoeba lugdunensis
Acanthamoeba polyphaga
87
70
76
0.02

hAT-NA3

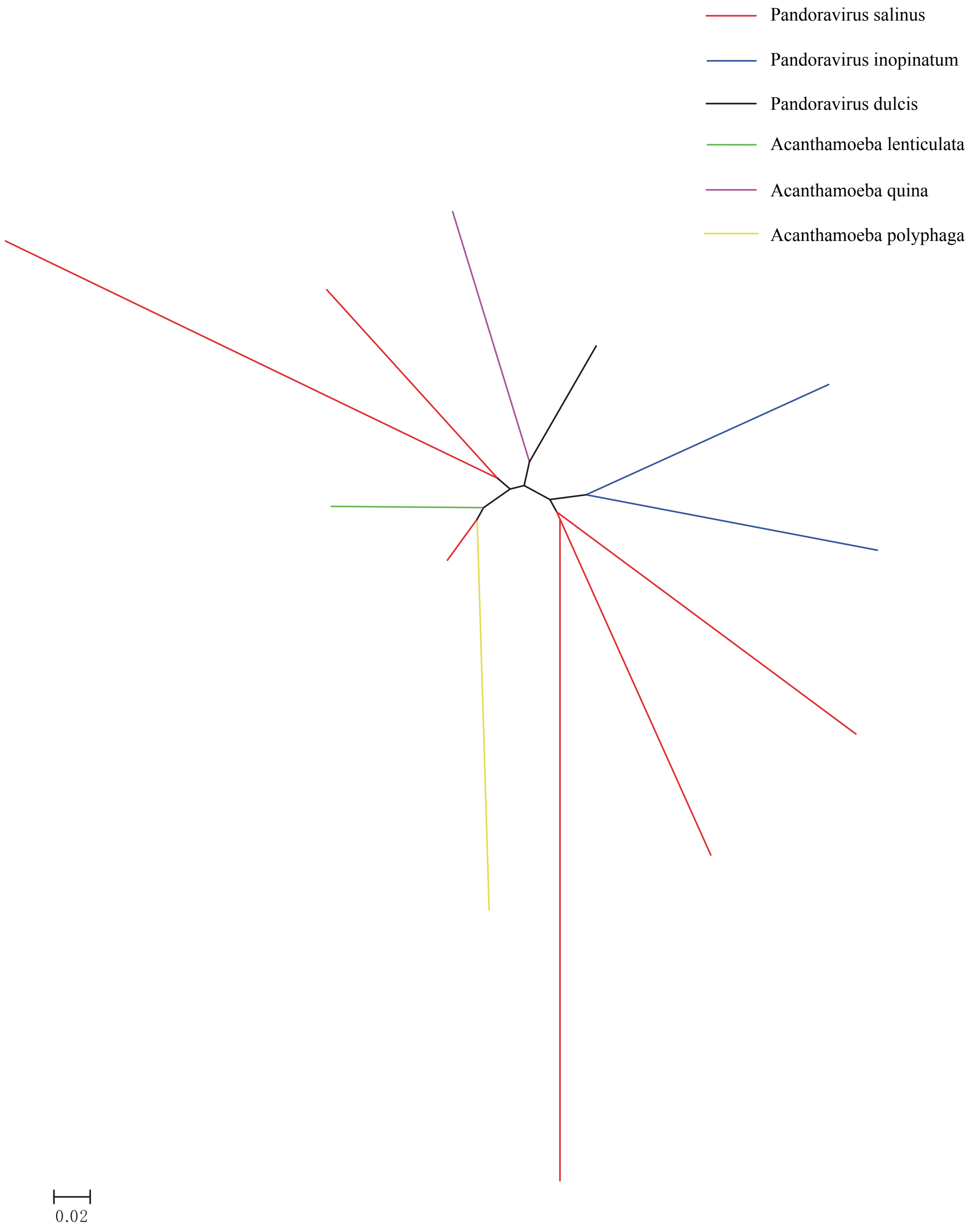

Pandoravirus salinus
Pandoravirus inopinatum
Pandoravirus dulcis
Acanthamoeba lenticulata
Acanthamoeba quina
Acanthamoeba polyphaga
0.02

hAT-NA4

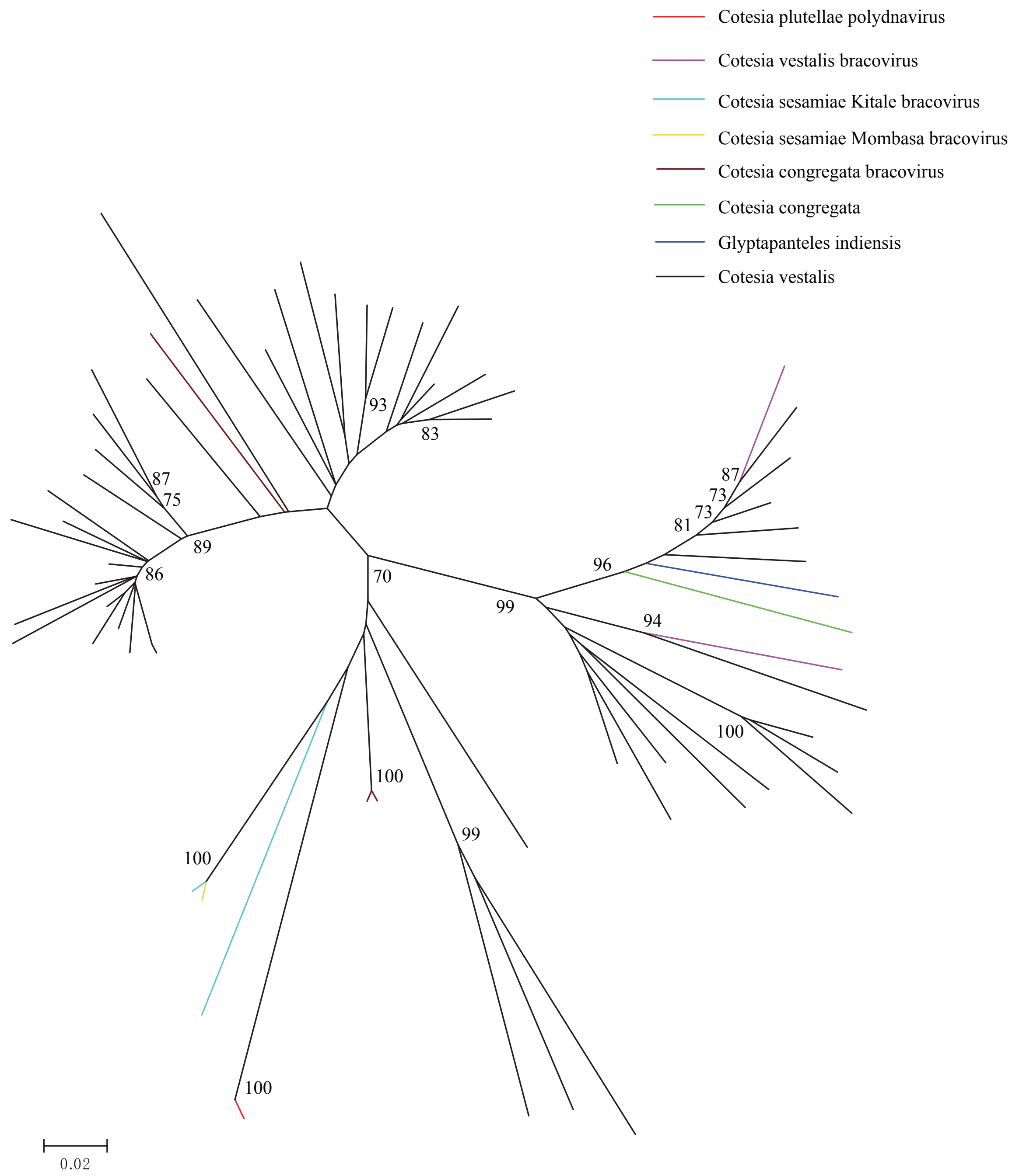
Cotesia plutellae polydnavirus
Cotesia vestalis bracovirus
Cotesia sesamiae Kitale bracovirus
Cotesia sesamiae Mombasa bracovirus
Cotesia congregata bracovirus
Cotesia congregata
Glyptapanteles indiensis
Cotesia vestalis
93
83
87
75
89
86
87
73
73
81
96
99
94
100
70
100
99
100
100
0.02

hATm-NA6
