# Supplementary material for: Unexpected invasion of miniature inverted-repeat transposable elements in viral genomes
Source: Mob DNA. 2018 Jun 18;9:19. doi: 10.1186/s13100-018-0125-4 (PMC6004678; doi:10.1186/s13100-018-0125-4)
Supplement: Supplementary file 12 — Table S5. Number of significant hits of hATm-6-RP retrieved using BlastP and TBlastN Tools in NCBI and the percent average identity find with the query. (DOC 44 kb) [file 13100_2018_125_MOESM12_ESM.doc]

Additional file 12: Table S5 Number of significant hits of *hATm-6-RP* retrieved using BlastP and TBlastN Tools in NCBI and the percent average identity find with the query

| Species | BlastP | | TBlastN | |
| --- | --- | --- | --- | --- |
|  | Hits | Average identity | Hits | Average identity |
| *Glyptapanteles flavicoxis* | 1 | 50 | 1 | 57 |
| *Cyphomyrmex costatus* | 7 | 38 | 0 | 0 |
| *Acyrthosiphon pisum* | 1 | 43 | 1 | 43 |
| *Bactrocera cucurbitae* | 1 | 36 | 0 | 0 |
| *B. dorsalis* | 0 | 0 | 1 | 37 |
| *B. latifrons* | 0 | 0 | 2 | 35 |
| *Cotesia congregata bracovirus* | 2 | 32.5 | 1 | 38 |
| *C. sesamiae Kitale bracovirus* | 0 | 0 | 1 | 86 |
| *C. congregata* | 2 | 32 | 2 | 39.5 |
| *Trachymyrmex cornetzi* | 2 | 42.5 | 0 | 0 |
| *Helobdella robusta* | 19 | 32.6 | 16 | 31.6 |
| *Bemisia tabaci* | 1 | 34 | 1 | 33 |
| *Diachasma alloeum* | 3 | 39 | 8 | 39 |
| *Microplitis demolitor* | 2 | 43.5 | 5 | 41.2 |
| *Drosophila suzukii* | 1 | 35 | 1 | 35 |
| *Amyelois transitella* | 1 | 33 | 1 | 32 |
| *Culex quinquefasciatus* | 1 | 32 | 1 | 32 |
| *Daphnia magna* | 1 | 31 | 0 | 0 |
| *Hydra vulgaris* | 2 | 31 | 1 | 30 |
| *Rhagoletis zephyria* | 0 | 0 | 3 | 36.3 |
| *Aedes albopictus* | 0 | 0 | 1 | 31 |
| *Agrilus planipennis* | 0 | 0 | 1 | 37 |
