# Supplementary material for: Unexpected invasion of miniature inverted-repeat transposable elements in viral genomes
Source: Mob DNA. 2018 Jun 18;9:19. doi: 10.1186/s13100-018-0125-4 (PMC6004678; doi:10.1186/s13100-018-0125-4)
Supplement: Supplementary file 13 — Table S6. Detail information of significant hits of proteins related to hATm-6-RP retrieved using BlastP and TBlastN Tools in NCBI. (DOC 109 kb) [file 13100_2018_125_MOESM13_ESM.doc]

Additional file 13: Table S6 Detail information of significant hits of proteins related to *hATm-6-RP* retrieved using BlastP and TBlastN Tools in NCBI

| Name in NCBI | Accession number | Species | Corresponding transposons in Repbase | Name in this study |
| --- | --- | --- | --- | --- |
| **BlastP results** | | | | |
| conserved hypothetical protein | ACE75260.1 | *Glyptapanteles flavicoxis* | - | hATm-1_GF |
| ALC62_13306 | KYM96042.1 | *Cyphomyrmex costatus* | - | hATm-1_CC |
| ALC62_11109 | KYM98193.1 | *Cyphomyrmex costatus* | - | hATm-2_CC |
| ALC62_02431 | KYN06617.1 | *Cyphomyrmex costatus* | - | hATm-3_CC |
| ALC62_15995 | KYM93405.1 | *Cyphomyrmex costatus* | - | hATm-4_CC |
| ALC62_03109 | KYN05953.1 | *Cyphomyrmex costatus* | - | hATm-5_CC |
| Chondroitin proteoglycan-2 | KYM96046.1 | *Cyphomyrmex costatus* | - | hATm-6_CC |
| ALC62_01887 | KYN07150.1 | *Cyphomyrmex costatus* | - | hATm-7_CC |
| LOC107885924 | XP_016665180.1 | *Acyrthosiphon pisum* | hAT-4D_AP | hAT-4D_AP |
| NADH-quinone oxidoreductase subunit D | JAD12022.1 | *Bactrocera cucurbitae* | - | hATm-1_BC |
| CcBV_30.5b | CCQ71090.1 | *Cotesia congregata bracovirus* | - | hATm-1_CcBV |
| CcBV_30.5 | YP_184879.1 | *Cotesia congregata bracovirus* | - | hATm-1_CcBV |
| hypothetical protein | CAR82250.1 | *Cotesia congregata* | - | hATm-1_CCo |
| CcPL6.029 | CCQ71357.1 | *Cotesia congregata* | - | hATm-1_CCo |
| ALC57_17860 | KYN10013.1 | *Trachymyrmex cornetzi* | - | hATm-1_TC |
| ALC57_16486 | KYN11357.1 | *Trachymyrmex cornetzi* | - | hATm-1_TC |
| HELRODRAFT_160147 | XP_009015400.1 | *Helobdella robusta* | hATm-1_HR | hATm-1_HR |
| HELRODRAFT_181271 | XP_009028760.1 | *Helobdella robusta* | hATm-2_HR | hATm-2_HR |
| HELRODRAFT_175867 | XP_009021487.1 | *Helobdella robusta* | hATm-2_HR | hATm-2_HR |
| HELRODRAFT_169182 | XP_009013297.1 | *Helobdella robusta* | hATm-2_HR | hATm-2_HR |
| HELRODRAFT_175856 | XP_009021483.1 | *Helobdella robusta* | hATm-2_HR | hATm-2_HR |
| HELRODRAFT_167628 | XP_009011363.1 | *Helobdella robusta* | hATm-2_HR | hATm-2_HR |
| HELRODRAFT_168079 | XP_009012015.1 | *Helobdella robusta* | hATm-2_HR | hATm-2_HR |
| HELRODRAFT_171475 | XP_009016434.1 | *Helobdella robusta* | hATm-2_HR | hATm-2_HR |
| HELRODRAFT_165054 | XP_009029201.1 | *Helobdella robusta* | hATm-2_HR | hATm-2_HR |
| HELRODRAFT_164196 | XP_009027446.1 | *Helobdella robusta* | hATm-3_HR | hATm-3_HR |
| HELRODRAFT_178679 | XP_009025017.1 | *Helobdella robusta* | hATm-3_HR | hATm-3_HR |
| HELRODRAFT_171303 | XP_009016278.1 | *Helobdella robusta* | hATm-2_HR | hATm-2_HR |
| HELRODRAFT_160152 | XP_009015405.1 | *Helobdella robusta* | hATm-1_HR | hATm-1_HR |
| HELRODRAFT_163840 | XP_009025851.1 | *Helobdella robusta* | hATm-2_HR | hATm-2_HR |
| HELRODRAFT_173825 | XP_009018683.1 | *Helobdella robusta* | hATm-5_HRo | hATm-5_HRo |
| HELRODRAFT_174241 | XP_009019032.1 | *Helobdella robusta* | hATm-2_HR | hATm-2_HR |
| HELRODRAFT_174188 | XP_009018988.1 | *Helobdella robusta* | hATm-2_HR | hATm-2_HR |
| HELRODRAFT_182127 | XP_009030672.1 | *Helobdella robusta* | hATm-2_HR | hATm-2_HR |
| HELRODRAFT_171365 | XP_009016337.1 | *Helobdella robusta* | hATm-2_HR | hATm-2_HR |
| LOC109041369 | XP_018913254.1 | *Bemisia tabaci* | - | hATm-1_BeT |
| LOC107046530 | XP_015124637.1 | *Diachasma alloeum* | - | hATm-2_DiA |
| LOC107044639 | XP_015122074.1 | *Diachasma alloeum* | - | hATm-1_ DiA |
| LOC107040428 | XP_015116001.1 | *Diachasma alloeum* | - | hATm-1_ DiA |
| LOC103578557 | XP_014296614.1 | *Microplitis demolitor* | - | hATm-1_MD |
| LOC106694182 | XP_014300045.1 | *Microplitis demolitor* | - | hATm-2_MD |
| LOC108019554 | XP_016942879.1 | *Drosophila suzukii* | - | hATm-1_DS |
| LOC106133808 | XP_013189132.1 | *Amyelois transitella* | - | hATm-1_AT |
| conserved hypothetical protein | XP_001867418.1 | *Culex quinquefasciatus* | - | hATm-1_CuQ |
| APZ42_000705 | KZS02306.1 | *Daphnia magna* | - | hATm-1_DaM |
| LOC101239659 | XP_012565401.1 | *Hydra vulgaris* | hATm-55_HM | hATm-55_HM |
| LOC100208603 | XP_012557288.1 | *Hydra vulgaris* | hATm-20_HM | hATm-20_HM |
| **TBlastN results** | | | | |
| clone BAC 16K15 | EF710649.1 | *Glyptapanteles flavicoxis* | - | hATm-1_GF |
| LOC107885924 | XP_016665180.1 | *Acyrthosiphon pisum* | hAT-4D_AP | hAT-4D_AP |
| LOC105226187 | XM_019990474.1 | *Bactrocera dorsalis* | - | hATm-1_BD |
| LOC108968290 | XM_018932209.1 | *Bactrocera latifrons* | - | hATm-1_BL |
| LOC108970106 | XM_018935257.1 | *Bactrocera latifrons* | - | hATm-2_BL |
| PL1 | HF586472.1 | *Cotesia congregata bracovirus* | - | hATm-1_CcBV |
| clone BAC 4C14 | EF710630.1 | *Cotesia sesamiae Kitale bracovirus* | - | hATm-1_CsBV |
| LEF-8 and HzNVORF128-like region | FM212912.1 | *Cotesia congregata* | - | hATm-1_CCo |
| *C. congregata* sequence | HF586477.1 | *Cotesia congregata* | - | hATm-1_CCo |
| hypothetical protein partial mRNA | XM_009017152.1 | *Helobdella robusta* | hATm-1_HR | hATm-1_HR |
| CH306-1A12 | AC171130.2 | *Helobdella robusta* | hATm-1_HR | hATm-1_HR |
| hypothetical protein partial mRNA | XM_009030512.1 | *Helobdella robusta* | hATm-2_HR | hATm-2_HR |
| hypothetical protein partial mRNA | XM_009023239.1 | *Helobdella robusta* | hATm-2_HR | hATm-2_HR |
| hypothetical protein partial mRNA | XM_009023235.1 | *Helobdella robusta* | hATm-2_HR | hATm-2_HR |
| hypothetical protein partial mRNA | XM_009015049.1 | *Helobdella robusta* | hATm-2_HR | hATm-2_HR |
| hypothetical protein partial mRNA | XM_009013767.1 | *Helobdella robusta* | hATm-2_HR | hATm-2_HR |
| hypothetical protein partial mRNA | XM_009013115.1 | *Helobdella robusta* | hATm-2_HR | hATm-2_HR |
| hypothetical protein partial mRNA | XM_009018186.1 | *Helobdella robusta* | hATm-2_HR | hATm-2_HR |
| hypothetical protein partial mRNA | XM_009030953.1 | *Helobdella robusta* | hATm-2_HR | hATm-2_HR |
| hypothetical protein partial mRNA | XM_009029198.1 | *Helobdella robusta* | hATm-3_HR | hATm-3_HR |
| hypothetical protein partial mRNA | XM_009026769.1 | *Helobdella robusta* | hATm-3_HR | hATm-3_HR |
| hypothetical protein partial mRNA | XM_009018030.1 | *Helobdella robusta* | hATm-2_HR | hATm-2_HR |
| hypothetical protein partial mRNA | XM_009027603.1 | *Helobdella robusta* | hATm-2_HR | hATm-2_HR |
| hypothetical protein partial mRNA | XM_009020435.1 | *Helobdella robusta* | hATm-5_HRo | hATm-5_HRo |
| hypothetical protein partial mRNA | XM_009032424.1 | *Helobdella robusta* | hATm-2_HR | hATm-2_HR |
| LOC109041369 | XP_018913254.1 | *Bemisia tabaci* | - | hATm-1_BeT |
| LOC107044639 | XM_015266588.1 | *Diachasma alloeum* | - | hATm-1_DiA |
| LOC107040428 | XP_015116001.1 | *Diachasma alloeum* | - | hATm-1_DiA |
| LOC107046530 | XP_015124637.1 | *Diachasma alloeum* | - | hATm-2_DiA |
| LOC107046530 | XM_015269153.1 | *Diachasma alloeum* | - | hATm-2_DiA |
| LOC107046530 | XM_015269154.1 | *Diachasma alloeum* | - | hATm-2_DiA |
| LOC107046530 | XM_015269155.1 | *Diachasma alloeum* | - | hATm-2_DiA |
| LOC107046530 | XM_015269151.1 | *Diachasma alloeum* | - | hATm-1_DiA |
| LOC107044806 | XM_015266845.1 | *Diachasma alloeum* | - | hATm-3_DiA |
| LOC103578557 | XP_014296614.1 | *Microplitis demolitor* | - | hATm-1_MD |
| LOC103572268 | XM_014443491.1 | *Microplitis demolitor* | - | hATm-3_MD |
| LOC103572268 | XM_014443492.1 | *Microplitis demolitor* | - | hATm-3_MD |
| LOC103572268 | XM_008550792.1 | *Microplitis demolitor* | - | hATm-3_MD |
| LOC106694182 | XP_014300045.1 | *Microplitis demolitor* | - | hATm-2_MD |
| LOC108019554 | XP_016942879.1 | *Drosophila suzukii* | - | hATm-1_DS |
| LOC106133808 | XP_013189132.1 | *Amyelois transitella* | - | hATm-1_AT |
| conserved hypothetical protein | XM_001867383.1 | *Culex quinquefasciatus* | - | hATm-1_CuQ |
| LOC101239659 | XP_012565401.1 | *Hydra vulgaris* | hATm-55_HM | hATm-55_HM |
| LOC108365609 | XM_017619674.1 | *Rhagoletis zephyria* | - | hATm-1_RZ |
| LOC108365609 | XM_017619675.1 | *Rhagoletis zephyria* | - | hATm-1_RZ |
| LOC108365609 | XM_017619676.1 | *Rhagoletis zephyria* | - | hATm-1_RZ |
| LOC109422465 | XM_019697246.1 | *Aedes albopictus* | - | hATm-1_AAl |
| LOC108745127 | XR_001939061.1 | *Agrilus planipennis* | - | hATm-1_AgP |
