# Supplementary material for: Unexpected invasion of miniature inverted-repeat transposable elements in viral genomes
Source: Mob DNA. 2018 Jun 18;9:19. doi: 10.1186/s13100-018-0125-4 (PMC6004678; doi:10.1186/s13100-018-0125-4)
Supplement: Supplementary file 14 — Table S7. The detail information of primers to amplify MITEs and their potential autonomous partner in viruses. (DOC 43 kb) [file 13100_2018_125_MOESM14_ESM.doc]

Additional file 14: Table S7 The detail information of primers to amplify MITEs and their potential autonomous partner in viruses

| Targeted MITEs | Primers | PCR | Sequencing |
| --- | --- | --- | --- |
| *hAT-NA1_PI* | Forward: CCGGATCATCTTTGTGGCAG  Reverse: TTTTGTCGCCGTCACTTGTG | + | + |
| *hAT-NA2_PI* | Forward: CGAGGTAAAGGCAGTGTGTG  Reverse: GTGGCGCTCATCTCTCTTTC | + | + |
| *hAT-NA3_PI* | Forward: CCGCAACCAATGGATTCCTC  Reverse: CCGGTCAATGATGCTCGATG | + | + |
| *hAT-NA4_PI* | Forward: CTTTACCTACACGCCGCAAG  Reverse: AATGTAAGCGCGACAGTGTC | + | + |
| *hAT-NA5_PI* | Forward: CGCCACGACCATCACTATTG  Reverse: CCTGTGCTTGCGGGTATTAA | + | + |
| *hATm-6_CcBV* | Forward: GTGTCCATGATCGATGCGTA  Reverse: TAAGCAAGGATCCAGAGCGG | + | + |
| *Submariner-NA_PI* | Forward: CGATACGTCAGCAATGGAGC  Reverse: AACGCACGACTGTCTAATGC | + | + |
| *CMC-NA_1_GfIV-1* | Forward: GGCTCGTCTCCGTCATCGTC  Reverse: TTGGTGCTCTTATATTAACGGAATATTAACGAGACACA | + | + |
| *CMC-NA_1_GfIV-2* | Forward: GTGCGTTCCTCAAATCGTCTTGGAC  Reverse: TGTGTATGCTGGATGGAAGGATTGACA | + | + |
| *CMC-NA_1_GfIV-3* | Forward: GCGGTGGGTACTTGGTTCAGATCTA  Reverse: AGTATCCAAATGGCCAGTCAGATAGAGTGT | + | + |
| *CMC-NA_1_GfIV-4* | Forward: GAAGACCTCAGCATACCAGGTGACT  Reverse: GATGTGTCCGAATTAGCACTATTTCCAAACGA | + | + |
| *CMC-NA_1_GfIV-5* | Forward: GCTACATTCAATTGTCAAGAGCCAGTATGC  Reverse: TACAATTTCTTCTACACGGAGAGGAATACCGA | + | + |
